# Overexpression of β-Arrestins inhibits proliferation and motility in triple negative breast cancer cells

**DOI:** 10.1038/s41598-021-80974-6

**Published:** 2021-01-15

**Authors:** Saber Yari Bostanabad, Senem Noyan, Bala Gur Dedeoglu, Hakan Gurdal

**Affiliations:** 1 Biotechnology Institute of Ankara University, 06135 Ankara, Turkey; 2 Department of Medical Pharmacology, Faculty of Medicine, University of Ankara, 06230 Ankara, Turkey

**Keywords:** Cancer, Breast cancer, Molecular medicine, Cell growth

## Abstract

β-Arrestins (βArrs) are intracellular signal regulating proteins. Their expression level varies in some cancers and they have a significant impact on cancer cell function. In general**,** the significance of βArrs in cancer research comes from studies examining GPCR signalling. Given the diversity of different GPCR signals in cancer cell regulation, contradictory results are inevitable regarding the role of βArrs. Our approach examines the direct influence of βArrs on cellular function and gene expression profiles by changing their expression levels in breast cancer cells, MDA-MB-231 and MDA-MB-468. Reducing expression of βArr1 or βArr2 tended to increase cell proliferation and invasion whereas increasing their expression levels inhibited them. The overexpression of βArrs caused cell cycle S-phase arrest and differential expression of cell cycle genes*, CDC45, BUB1, CCNB1, CCNB2, CDKN2C* and reduced HER3, IGF-1R, and Snail. Regarding to the clinical relevance of our results, low expression levels of βArr1 were inversely correlated with CDC45, BUB1, CCNB1, and CCNB2 genes compared to normal tissue samples while positively correlated with poorer prognosis in breast tumours. These results indicate that βArr1 and βArr2 are significantly involved in cell cycle and anticancer signalling pathways through their influence on cell cycle genes and HER3, IGF-1R, and Snail in TNBC cells.

## Introduction

β-Arrestins (βArrs) are adaptor or scaffolding proteins regulating the signalling pathways of G protein coupled receptors (GPCRs)^1^. Their role in desensitization, inhibition, switching, or trafficking of GPCRs signalling is particularly well documented^2^. The agonist stimulation of GPCR leads to its phosphorylation followed by the binding of βArrs to GPCR, which desensitizes and inhibits GPCR signalling^2–4^. This interaction not only inhibits GPCR signalling but also activates different GPCR pathways that are important for cellular growth and cancer development^5^. The binding of βArrs to phosphorylated GPCRs leads to clathrin dependent endocytosis and internalization of the GPCR-βArrs complex. This causes degradation or recycling of GPCRs and activation of the intracellular signalling molecules regulating cell proliferation and motility, such as MAPKs/ERK1/2, JNK, PI3K, and AKT^5–7^. Another important pathway for cellular growth and cancer is transactivation of growth factor receptors with GPCRs in which βArrs have a crucial role^7,8^. βArrs not only regulate the activity of GPCRs and G-proteins but also mediate signalling involving protein kinases (Src, Raf-1, Akt, ERK1/2, JNK) and transcription factors (MDM2, p53, IκB). These are significant modulators of cellular growth and cancer cell migration, invasion, and metastasis^9–12^.

There are some indications that βArrs may trigger malignant signalling and cancer progression through activation of protein kinases (Src, MAPKs, PI3K), growth factor receptors, and transcription factors^5,7,13^. Furthermore, βArrs affect cell survival and can prevent GPCR-induced apoptosis^14,15^.

On the other hand, some studies indicate that βArr-mediated signalling reduces cell proliferation, migration, invasion, and metastasis, and induces apoptosis and an anti-cancer response pattern in some types of cancer^16–18^. They can facilitate cell death by mediating apoptotic signalling^17,18^. Overexpression of βArr2 induces G1-phase cell cycle arrest and suppresses tumorigenicity in renal cell carcinoma^17^. While overexpression of βArr2 significantly reduces cancer cell migration and invasion, down-regulation of it promotes tumour invasion and indicates a poor prognosis for hepatocellular carcinoma^16^. Parallel to these results, depletion of βArr2 in the murine model of lung cancer significantly increased Lewis lung cancer tumour growth, angiogenesis, and metastasis^19^.

Differential expression of βArrs has been shown in several types of cancers, including lung, hepatocellular, colorectal, and breast cancers^16,19–21^. βArr1 expression decreases while βArr2 level increases during breast cancer progression. These changes also correlate with a poor clinical outcome^21^. In addition, stromal expression of βArr1 can predict clinical outcome and tamoxifen response in breast cancer^22^ while βArrs seem to be functionally significant in breast cancers as they regulate lysophosphatidic acid or constitutive-activated receptor-2 mediated cell migration and invasion in TNBCs^23^. βArr2 also inhibits opioid induced apoptosis through anti-apoptotic Akt and pro-apoptotic caspase-8 pathways in breast cancer cells^24^.

While βArrs may have varying effects on cancer cell regulation, these findings indicate overall that their expression and function are important, so further studies are needed to elucidate their role in breast cancers.

This study examined the influence of βArrs on the regulation of TNBC cells, MDA-MB-231, and MDA-MB-468. These cell lines are negative for estrogen receptor (ER-), progesterone receptor (PR-), human epidermal growth factor receptor 2 (HER2-), and are resistant to anti-cancer treatments. βArrs are the main regulators of GPCRs such as lysophosphatidic acid, protease activated, β-adrenergic, opioid, and angiotensin receptors, which mediate significant signalling in TNBCs^23–25^.

Therefore, to clarify the regulatory importance of βArrs, we changed their expression levels in MDA-MB-231 and MDA-MB-468 cells and evaluated cell proliferation, cell motility and cell cycle. To better understand the molecular mechanisms, microarray analysis was performed with βArr1 or βArr2 overexpressing MDA-MB-231 cells, and differentially expressed genes were identified. The genes that were enriched after pathway enrichment analysis, especially in cancer related pathways, were validated by qRT-PCR or Western-blot analyses. We also analysed TCGA data set comparing the expression level of βArr1 or βArr2 and cell cycle genes obtained from our studies to evaluate and discuss the clinical relevance of our results. The combination of mRNA profiling, bioinformatics analysis and experimental data suggested that cell cycle arrest and growth factor receptor downregulation were the main mechanisms regulating cancer cell behaviour in TNBC cells following βArrs overexpression.

## Methods

### Materials

The cell lines (MDA-MB-231, MDA-MB-468, SKBR3, BT-474) were obtained from the American Type Culture Collection (ATCC, Manassas, VA, USA). Human β-arrestin-1 siRNA (sc-29741), β-arrestin-2 siRNA (sc-29208), and scrambled oligo-ribonucleotide complex utilized as control (sc-37007), siRNA transfection reagent (sc-29528), and transfection medium (sc-36868) were all obtained from Santa Cruz Biotechnology. Human β-arrestin-1 (ARRB1) cDNA (SC303424), human β-arrestin-2 (ARRB2) cDNA (SC108950), TurboFectin 8.0 transfection reagent (TF81001), cloning vector PCMV6-XL6 (PCMV6XL6), and cloning vector PCMV6-XL5 (PCMV6XL5) were all purchased from OriGene Technologies, MD, USA. The antibodies, β-Arrestin-1 (sc-9182), β-Arrestin-2 (sc-13140), CDC45 (sc-55569, CCNB1 (sc-245), CCNB2 (sc-28303), BUB1 (sc-365685), GAPDH (1:5,000; Sc-166545), goat anti-rabbit (1:10,000; Sc-31460), rabbit anti-goat (1:10,000; Sc-2768), and donkey anti-mouse (1:10,000; Sc-2314) were obtained from Santa Cruz Biotechnology, CA, USA. β-Arrestin-1 (D803J), β-Arrestin-2 (C16D9), IGF-1R (1:1000; D23H3), E-Cadherin (1:1000; 24E10), Snail (1:1000; C15D3), and Vimentin (1:1000; D21H3) were obtained from Cell Signaling Technology, the Netherlands.

### Cell culture and transfection

MDA-MB-231 and MDA-MB-468 human triple negative breast cancer cells were maintained in Dulbecco’s modified Eagle’s medium (DMEM) and Eagle’s minimal essential medium (EMEM), supplemented with 10% fetal bovine serum and 1% penicillin/streptomycin at 5% CO_2_, 37 °C with 90–95% humidity. The cells (2.5 × 10^5^) were cultured in a 6-well plate and left for 24 h to reach 70–80% confluency for cDNA and siRNA transfections. DNA transfections were performed according to the manufacturer’s protocol using, for each well, 4 µl of TurboFectin Transfection 8.0 Reagent, 100 µl of Opti-MEM I, and 2 µg of control nontarget DNA (Cloning vector PCMV6XL5 or PCMV6XL6), and human βArr1cDNA or human βArr2 cDNA. siRNA transfections were performed according to the manufacturer’s protocol by using, for each well, 4 µl of siRNA-transfection reagent, 100 µl of transfection medium, and 1 µg of control siRNA, and human βArr1siRNA or human βArr2 siRNA. After transfection for 48 h at 37 °C, the medium was replaced, and the cells were cultured for an additional 12 h.

### Real time cell proliferation and invasion assay by impedance measurements

These assays were performed as described previously by using the xCelligence Real-Time Cell Analyzer (RTCA) DP system (ACEA Biosciences, Inc., San Diego, CA, USA), according to the manufacturer’s protocol. Briefly, the MDA-MB-231 or MDA-MB-468 cells (5000 cells in 150 µl medium/well; E-plate) were seeded into each well^26^. Subsequently, the impedance of each well of the E-plate was measured continuously for 72–96 h at 37 °C with 5% CO_2_. For the cell invasion assay, cells were incubated for 4 h in serum free medium and a total of 40,000–50,000 cells were seeded in the upper chamber of CIM-plates, which was coated with Matrigel solution (Corning, 354,234). FBS containing medium was added to the lower chamber of the CIM-plates. The impedance measurement was recorded for 40 h. The results were analysed using RTCA data analysis software 1.0 (ACEA Biosciences, Inc., San Diego, CA, USA). The cell proliferation and cell migration or invasion indices were calculated from 4–5 replicates of independent experiments.

### Cell cycle assays

Cell cycle assays were performed using Annexin V FITC (640906, BioLegend, San Diego, CA, USA), propidium iodide staining kit (411301, BioLegend, SanDiego, CA, USA) and NovoCyte Flow cytometer (ACEA Biosciences, Inc., San Diego, CA, USA) using NovoExpress 1.0.2 analysis software, according to each manufacturer’s protocols.

### Protein isolation and Western blotting

Protein isolation and Western blotting were carried out as described previously^27^. The cells in 6 well plates were immediately placed on ice, washed with ice-cold PBS, and homogenized in 100 μl lysis buffer (Roche Diagnostics GmbH, Mannheim, Germany) containing 1% Nonidet P40, 0.02 M sodium orthovanadate, and protease inhibitors. Following homogenization, the cells were incubated for 15 min and centrifuged at 5000×*g* for 5 min at 4 °C. After collecting the supernatant, the protein concentration was determined using the Bradford protein assay and stored at − 80 °C. Electrophoresis (depending on experiments, 20–60 μg protein/per lane) was performed on newly cast 8–10% sodium dodecyl sulphate (SDS)-polyacrylamide gels followed by transfer onto polyvinylidene difluoride membranes. After transfection, we cut the blot membranes according to molecular weight marker and performed hybridisation with different antibodies to be able to obtain multiple protein bands from one blot membrane. Because of this, we could not provide full length of images of some of the western-blot in the supplementary material file. The membranes were blocked for 2 h at 22 °C in PBS with 20 mM NaH_2_PO_4_–Na_2_HPO_4_ (pH 7.6) containing 154 mM NaCl, 5% non-fat dry milk and 0.1% Tween-20. The membranes were incubated with the appropriate primary antibodies overnight at 4 °C and washed three times for 10 min with TBS-0.2% Tween-20 prior to incubation for 1 h at 22 °C with horseradish peroxidase conjugated anti-rabbit, anti-mouse, or anti-goat secondary antibody. Following washing, the membranes were soaked in Clarity Western ECL Substrate (Bio-Rad Laboratories, Inc., Hercules, CA, USA) and imaged using a ChemiDoc MP Imaging System (Bio-Rad Laboratories, Inc.). Band intensities were quantified using Image Lab software (version 5; Bio-Rad Laboratories, Inc.).

### Microarray analysis

Microarray analysis was performed as described previously^28^. For the RNA isolation, the cells were put into Trizol reagent (AppliChem, Darmstadt, Germany), disrupted with a homogenizer, and total RNA was isolated according to the manufacturer’s instructions. DNase treatment was performed on each RNA after RNA isolation by using DNase I (EN0521; Thermo Scientific). The concentration of isolated RNA and absorbance ratio at 260 nm to 280 nm were measured with a NanoDrop ND-1000 spectrophotometer (NanoDrop Technologies, Montchanin, DE, USA). The genome wide gene expression profile was evaluated using a GeneChip Human Genome U133 Plus 2.0 Array (Affymetrix, Santa Clara, CA). One microgram of total RNA was processed using the protocols and instruments recommended by the manufacturer.

### Microarray data analysis

Two biological replicates were used for each βArr1-cDNA and βArr2-cDNA transfected and control-cDNA transfected MDA-MB-231 cell. A total of 6 samples were analysed. BRB Array Tools 4.3.2. stable release was used for the normalization and statistical analysis^29^. Bioconductor packages were used for normalization and statistical comparisons. The data were normalized by Quantile normalization method^30^. The statistical comparisons were performed using the t-test based class comparison function of BRB Array Tools, “between group of arrays (BGA)”. In determining the differential expression of mRNAs between βArr1 and βArr2 overexpressed and control cells, a *p* value less than 0.05 and fold changes more than 2 were used as cut-off values. The complete array data can be found in GEO with the accession number GSE156802.

A separate two-way hierarchical clustering analysis was performed for the clustering of samples according to both genes and sample types using Cluster 3.0^31^. The heatmaps were visualized by Treeview^30^. Common differentially expressed genes between βArr1 and βArr2 overexpressed MDA-MB-231 cells were identified using a Venn diagram drawing tool, VENNY^32^.

### Validation of microarray results by real time quantitative RT-PCR (qRT-PCR)

qRT-PCR was performed as described previously^33^. First strand cDNA was synthesized using 1 µg total RNA with the iScript cDNA Synthesis Kit according to the manufacturer’s instructions (Bio-Rad, Germany). In a reverse-transcription reaction, polyadenylated mRNAs were converted into cDNA by reverse transcriptase with oligo-dT priming. The cDNAs were then used for qRT-PCR profiling. The cDNA was diluted 1:5 before being used as a PCR template and stored at − 20 °C until further use.

The qRT-PCR analysis was performed on a Roche LightCycler 480 using primers specific to the target genes and the SYBR Green Master Mix (Roche, Germany). The amplification mixtures contained 1.0 µl 1:5 diluted cDNA, 5.0 µl SYBR Green PCR Master Mix, 1.0 µl from each forward and reverse primer, and 2 µl RNase-free water in a total volume of 10 µl. Cycling conditions were as follows: 95 °C for 15 min for initial activation and then 40 cycles of 94 °C for 15 s, 55 °C for 30 s, and 70 °C for 30 s during amplification. The ΔΔCt method was used to analyze the qRT-PCR results and GAPDH was used as the normalization factor^33^.

### Statistical and bioinformatics analysis

#### TCGA data analysis

The expression levels of the two βArrs in the clinical samples were assessed in breast tumour tissues, normal samples, and PAM50^34^ subtypes using the TCGA Breast Cancer BRCA data set (*n* = 1247) through the Xena Browser^35^. The BRCA data from TCGA was downloaded and individual data graphs were plotted for tumour/normal comparisons and for PAM50 classification of breast cancer both for ARRB1, ARRB2, CCNB1,CCNB2, BUB1 and CDC45 gene expression by using statistical analysis tool Minitab Software (Minitab LLC, State College, Pennsylvania, USA). The data was reanalysed statistically and significance of the differences between the groups were indicated as p values.

#### Pathway enrichment analysis of the target genes

To better understand the functional characteristics of differentially expressed mRNAs, a KEGG pathway enrichment analysis was performed using the WebGestalt tool^36^. As the default setting, the minimum number of genes was adjusted to 2 from the list required for a pathway to be considered. The adjusted *p* value of each enriched pathway was calculated following Benjamini and Hockberg^37^ and the statistically enriched pathways were obtained using a hypergeometric test (*p* value < 0.05).

Data are reported as means ± standard error of the mean, while ‘n’ represents the number of independent experiments for each indicated condition. Comparisons between groups were performed with *Student’s t* test or one-way analysis of variance followed by Tukey’s post-hoc test, with *p* < 0.05 considered to indicate a statistically significant difference.

### Ethics approval

This article does not contain any studies with human participants or animals performed by any of the authors.

## Results

### βArr1 or βArr2 are regulated in breast tumours

**Figure 1:**
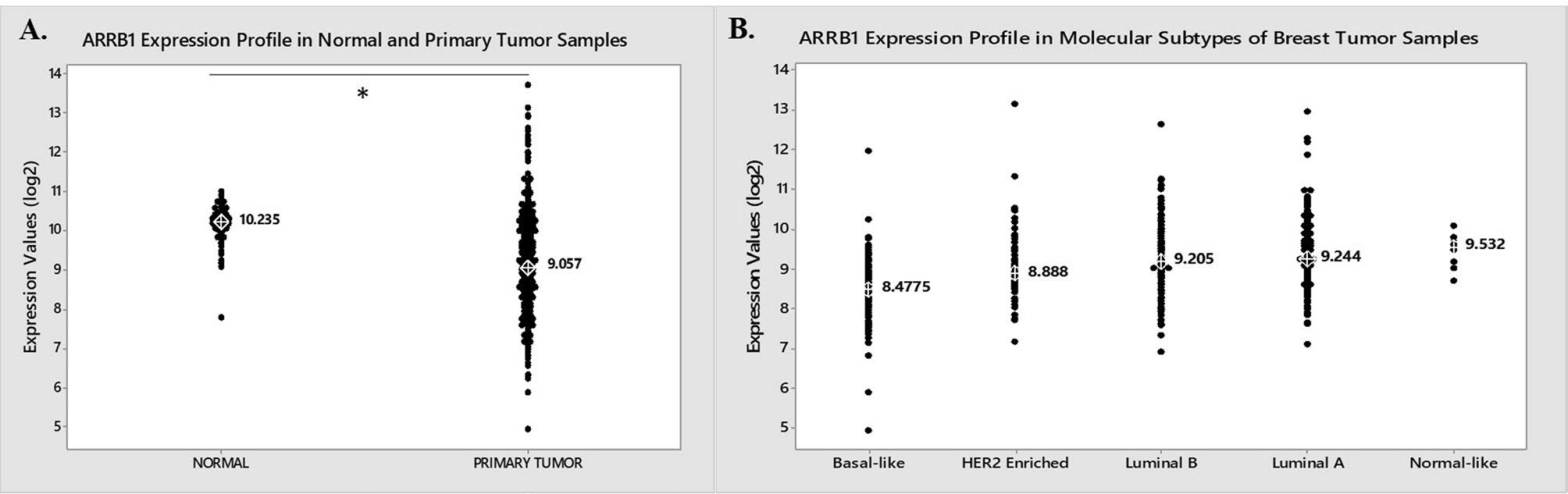
TGCA data analysis and downregulation of βArr1 expression in tumour samples. (**A**) The expression levels of βArr1 (ARRB1) in the clinical samples were assessed in breast tumour tissues and normal samples using the TCGA Breast Cancer BRCA data set (*n* = 1211). The expression profiles of ARRB1 gene in 114 normal samples and 1097 primary tumour samples obtained from TCGA are shown in individual value plots. The median expression values are marked for each sample type, which is 10.235 for normal samples and 9.057 for the tumour samples. ARRB1 gene is significantly downregulated in tumour samples compared to normal samples (t-test; *p* = 1.35977E^−33^). (**B**) The expression profiles of ARRB1 gene in 98 Basal-like, 58 HER2-enriched, 127 Luminal B, 231 Luminal A and 8 Normal-like breast tumour samples obtained from TCGA are shown in individual value plots. The values given on the value plots are the median values for each sample type. According to ANOVA and Tukey’s post-hoc test result ARRB1 expression is significantly downregulated in Basal-like tumours compared to Normal-like, Luminal A, Luminal B or HER2-enriched subtype (ANOVA; *p < 0.002).

To determine the clinical significance of the two βArrs we explored the expression profiles of these two genes in TCGA breast tissue data set^35^. The TCGA data analysis showed that βArr1 expression was downregulated significantly in breast tumours compared to normal tissues (Fig. 1A). Further analysis performed with the molecular subtypes of breast cancer (Normal-like, Luminal A, Luminal B, HER2 Enriched and Basal-like) also showed gradual downregulation of βArr1 from Normal-like tumours to the most severe subtype of breast cancer, Basal-like ones (Fig. 1B). The downregulation was statistically significant in Basal-like tumours compared to Normal-like, Luminal A, Luminal B or HER2-enriched subtypes (ANOVA; *p* < 0.002) (Fig. 1B). The expression difference among the other subtypes was not statistically different. (ANOVA; *p* > 0.05) (Fig. 1B). A similar analysis performed for βArr2 showed a slight increase in the gene expression in tumour samples compared to normal samples (Supplementary Fig. 1A) (ANOVA; *p* < 0.027). On the other hand, the gene was downregulated significantly in Luminal A, Luminal B or Her2 Enriched compared to the Normal-like or Basal-like breast tumours (Supplementary Fig. 1B) (ANOVA; *p < 0.0001). βArr2 expression is not significantly different between Normal-like and Basal-like breast tumours (ANOVA; *p* > 0.05).

**Figure 2:**
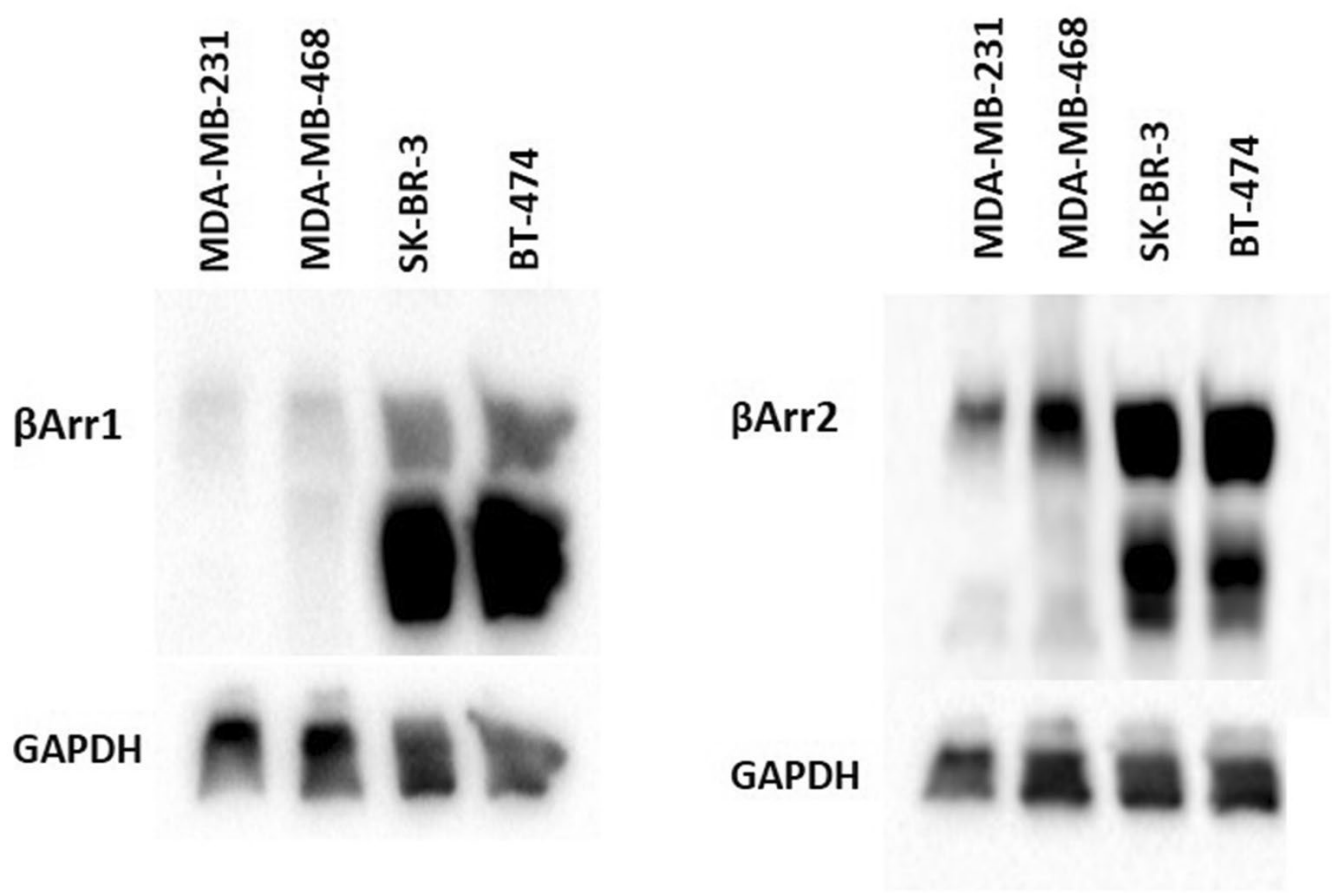
Expression of βArr1 or βArr2 in breast cancer cell lines. Representative Western-blot analysis of the expression of βArr1 or βArr2 from the cell lysate (30 µg/per lane) of the most aggressive breast cancer cell lines TNBCs (MDA-MB-231 and MDA-MB-468) and the less aggressive ones Her2-enriched (SKBR3) and Luminal B (BT474) was shown. The analysis showed a single band in MDA-MB-231 and MDA-MB-468 and two bands in SKBR3 and BT474. The second lower band in western-blot analysis in SKBR3 and BT474 is most probably related to splice variants of βArr1 and βArr2. The expression of both βArr1 and βArr2 was significantly less in TNBC cells (MDA-MB-231 and MDA-MB-468) than Her2-enriched, SKBR3, or Luminal B, BT474. The expression of βArr1 and βArr2 was significantly less in MDA-MB-231 than in MDA-MB-468.

In addition to TCGA data, to show the variability of the expression of βArr1 or βArr2 in the most aggressive breast cancer cell lines, TNBC cells (MDA-MB-231 and MDA-MB-468) and the less aggressive ones Her2-enriched (SKBR3) and Luminal B (BT474), Western-blot analysis was performed (Fig. 2).

The expression of both, βArr1 and βArr2, was significantly less in TNBC cells (MDA-MB-231 and MDA-MB-468) than SKBR3 or BT474 (Fig. 2). We observed a second lower band in Western-blot analysis in SKBR3 and BT474. This second band is most probably related to splice variants of βArr1 and βArr2, which have been shown previously^38^. The expression of βArr1 and βArr2 was also different and less in MDA-MB-231 than in MDA-MB-468 (Fig. 2).

### Regulation of βArr1 or βArr2 expression levels modulates cell proliferation in TNBC cells

**Figure 3:**
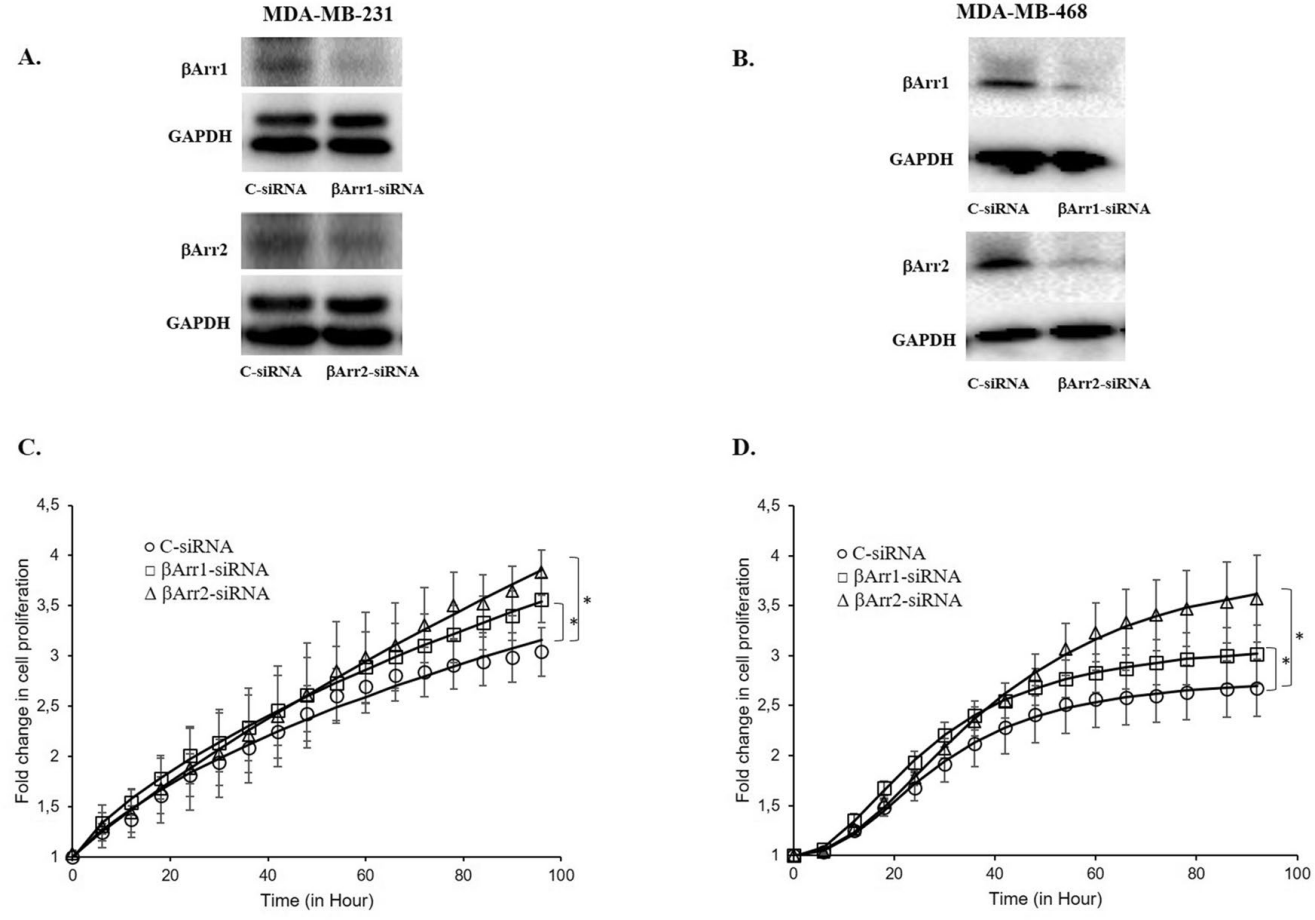
Silencing expression of βArr1 or βArr2 and increased cell proliferation. The expression level of βArr1 or βArr2 was reduced by transfecting βArr1 or βArr2-siRNA to MDA-MB-231 or MDA-MB-468 cells and (**A**) and (**B**) show a significant decline in the level of these proteins determined by Western-Blot analysis. (**C**) and (**D**) show impedance measurement of cell proliferation with real time cell electronic sensing system in C (control-siRNA)-, βArr1-, or βArr2-siRNA transfected MDA-MB-231 and MDA-MB-468 cells. Cell proliferation was recorded continuously just after seeding cells. The cell proliferation after four hours was defined as the baseline and the fold change of the baseline was determined for further measurements and shown as the fold change in cell proliferation in C and D. Reducing the expression level of βArr1 or βArr2 increased the cell proliferation in both cell lines. Data are presented as mean ± standard error of the mean; n = 4–5; ^*^ = *p* < 0.05 versus C-siRNA.

**Figure 4:**
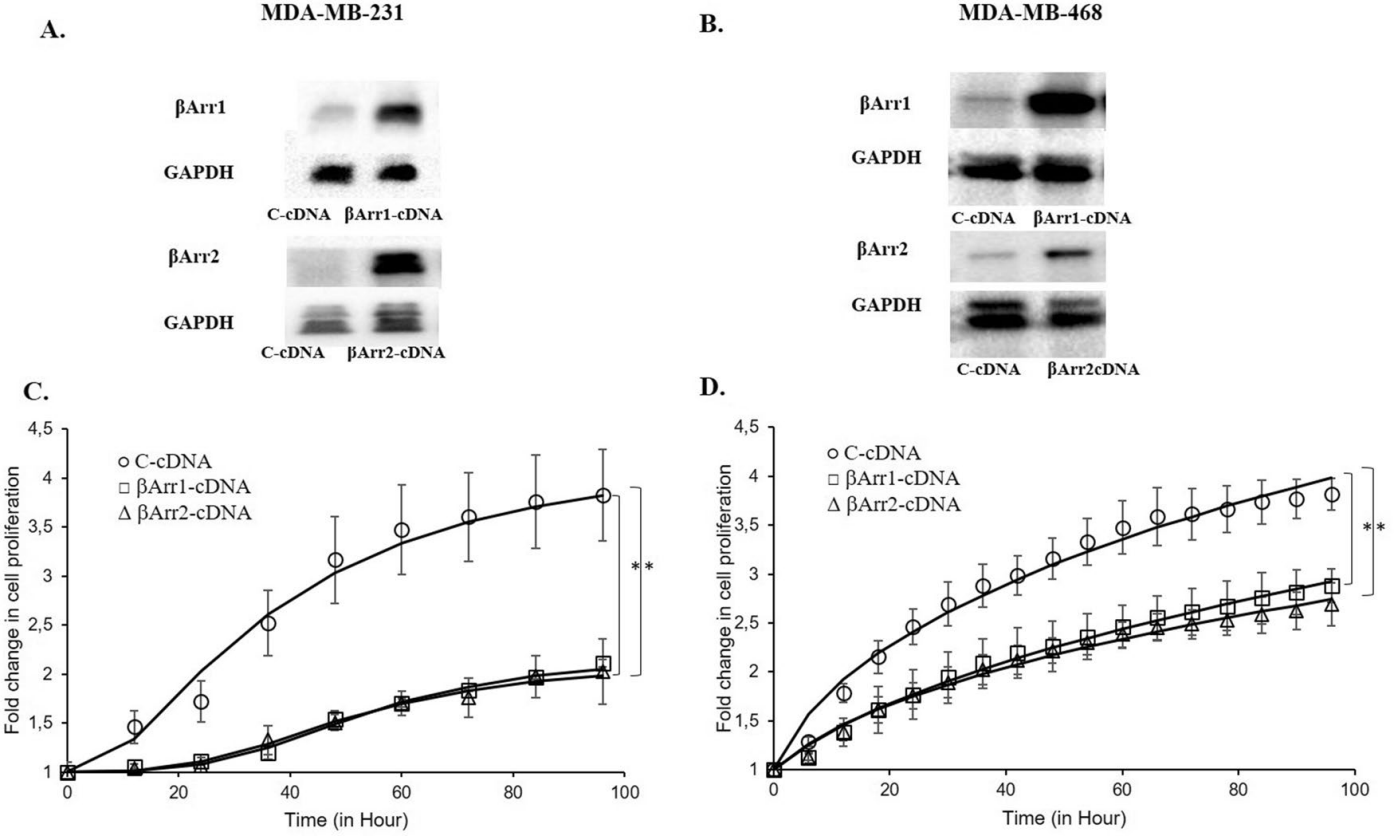
Overexpression of βArr1 or βArr2 and decreased cell proliferation. The expression level of βArr1 or βArr2 was increased by transfecting βArr1 or βArr2-cDNA to MDA-MB-231 or MDA-MB-468 cells and (**A**) and (**B**) show a significant increase in the level of these proteins determined by Western-blot analysis. (**C**) and (**D**) show impedance measurement of cell proliferation with real time cell electronic sensing system in C (control)-, βArr1-, or βArr2-cDNA transfected MDA-MB-231 and MDA-MB-468 cells. Cell proliferation was recorded continuously just after seeding cells. The cell proliferation after four hours was defined as the baseline and the fold change of the baseline was determined for further measurements and shown as the fold change in cell proliferation in C and D. Overexpression of βArr1 or βArr2 significantly inhibited the cell proliferation in both cell lines. Data are presented as mean ± standard error of the mean; n = 4–5; ^*^ = *p* < 0.05 versus C-cDNA.

Transfection of βArr1 and βArr2-siRNA significantly reduced their expression levels in MDA-MB-231 and MDA-MB-468 cells (Fig. 3A,B). Concordant with the TCGA data, downregulation of βArr1 and βArr2 increased the proliferation capacity of both TNBC cells, MDA-MB-231, and MDA-MB-468 (Fig. 3C,D). Conversely, transfection of βArr1 and βArr2-cDNA significantly increased their expression levels in MDA-MB-231 and MDA-MB-468 (Fig. 4A,B) and reduced the proliferation capacity of MDA-MB-231 and MDA-MB-468 cells compared to control cells (Fig. 4C,D).

### Microarray analysis

**Figure 5:**
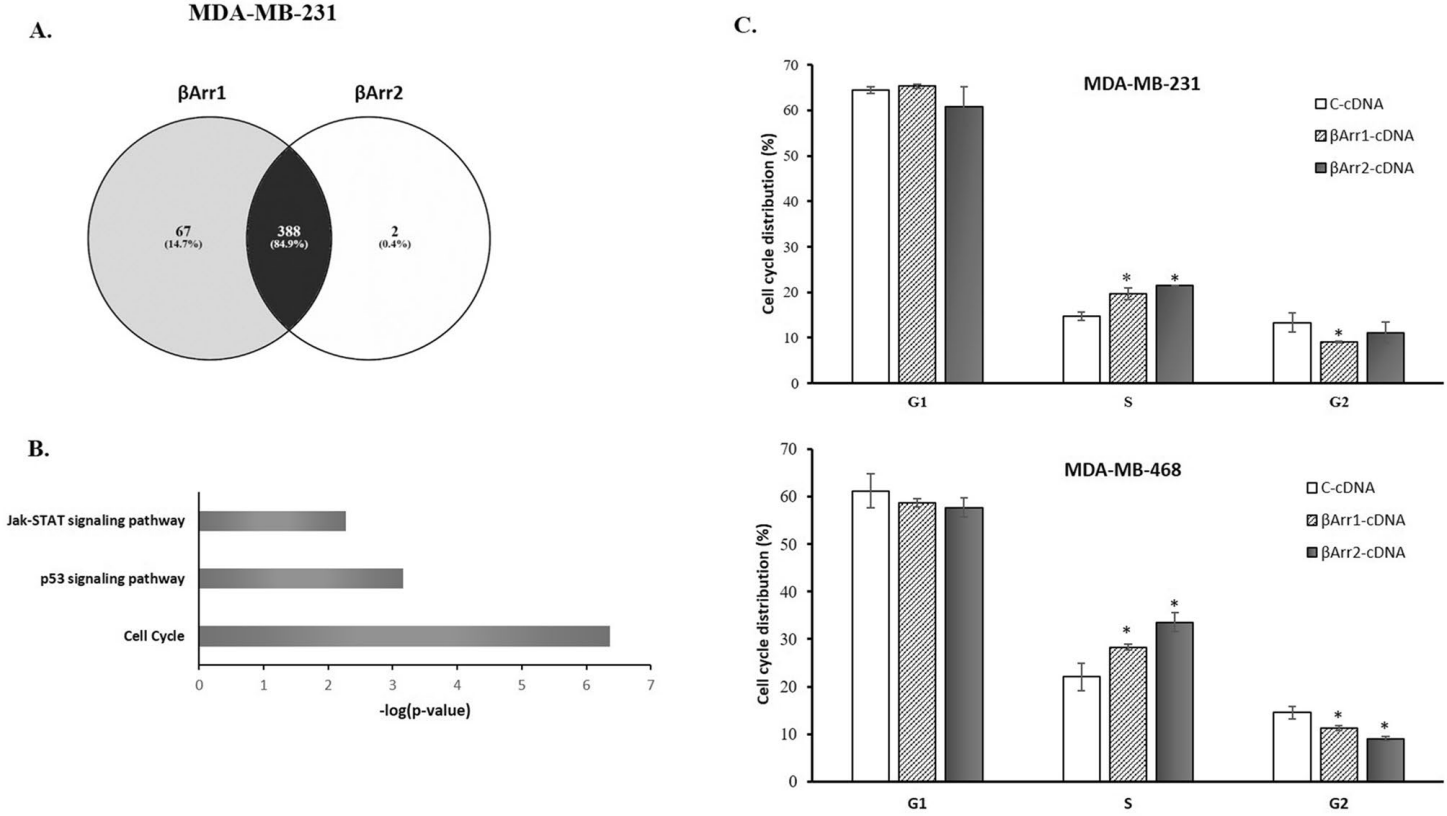
Microarray analysis revealed the cell cycle. Microarray and cell cycle analysis were performed in C (control)-, βArr1-, or βArr2-cDNA transfected MDA-MB-231 cells. Cell cycle analysis was also performed in C (control)-, βArr1-, or βArr2-cDNA transfected MDA-MB-468 cells. (**A**) Microarray data analysis showed that 455 and 390 genes were differentially expressed in βArr1 and βArr2-overexpressed MDA-MB-231 cells respectively compared to control cells. VENNY analysis conducted with differentially expressed genes showed that of these differentially expressed genes, 388 were common to both βArr1 and βArr2 overexpressing MDA-MB-231 cells, including almost all the differentially expressed genes in βArr2 overexpression. (**B**) The pathway enrichment and the KEGG pathway analysis were performed with these differentially expressing genes. These genes were significantly enriched in cancer related pathways, Jak-Stat, p53 signalling pathway and cell cycle genes. (**C**) Cell cycle analysis showed that S-phase cell populations significantly increased following βArr1 and βArr2 overexpression in MDA-MB-231 and MDA-MB468 cells. Eventually, cells in G2-phase decreased in both cell lines. Data are presented as mean ± standard error of the mean; n = 4–5; ^*^ = *p* < 0.05 versus C-cDNA.

To further characterize the molecular functions of these two genes in TNBC, microarray analysis was performed with the MDA-MB-231 cells transfected with βArr1-cDNA, βArr2-cDNA, and control-cDNA. This showed that 455 and 390 genes were differentially expressed in βArr1 and βArr2-overexpressed MDA-MB-231 cells respectively compared to control cells. Furthermore, Venny Venn’s diagram drawing tool was used to find out the common differentially expressed genes between βArr1 and βArr2 overexpressed cells. Of these differentially expressed genes, 388 were common to both βArr1 and βArr2, including almost all the differentially expressed genes in βArr2 overexpression (Fig. 5A). To understand the molecular functions of these genes, pathway enrichment analysis was performed and the KEGG pathways were identified via Webgestalt, WEB-based GEneSeTAnaLysis Toolkit^36^. The differentially expressed genes were significantly enriched in cancer related pathways, such as the cell cycle, p53, and Jak-STAT signalling pathways (Fig. 5B). Since the most significant pathway was the cell cycle, we focused on this pathway and the genes that are the key players in it.

### Overexpression of βArr1 or βArr2 arrests the cells in S-phase

Although we only conducted the microarray analysis with MDA-MB-231 cells, we performed all the further assays with MDA-MB-231 and MDA-MB-468 cells. The cell cycle analysis showed that S and G2 phase fractions in control cDNA transfected MDA-MB-231 cells were 15% and 13% respectively. These values changed significantly following overexpression of βArr1 or βArr2 in MDA-MB-231 cells, with S phase fractions increasing respectively to 20% and 22%, and G2 phase fractions declining to 9% and 11% (Fig. 5C). Similarly, in control cDNA transfected MDA-MB-468 cells, S and G2 phase fractions were 22% and 14% respectively. Following overexpression of βArr1 or βArr2, S phase fractions increased to 28% and 33% and G2 phase fractions declined to 11% and 9% respectively (Fig. 5C). Overexpression of both genes caused significant S-phase arrest in both triple negative cell lines.

The regulatory roles of βArr1 or βArr2 in the cell cycle found by microarray and pathway enrichment analysis were also validated by cell cycle analysis. Cells were arrested in S-phase when either βArr1 or βArr2 were overexpressed in both cell lines, MDA-MB-231 and MDA-MB-468.

### βArr1 and βArr2 regulated cell cycle pathway genes

**Figure 6:**
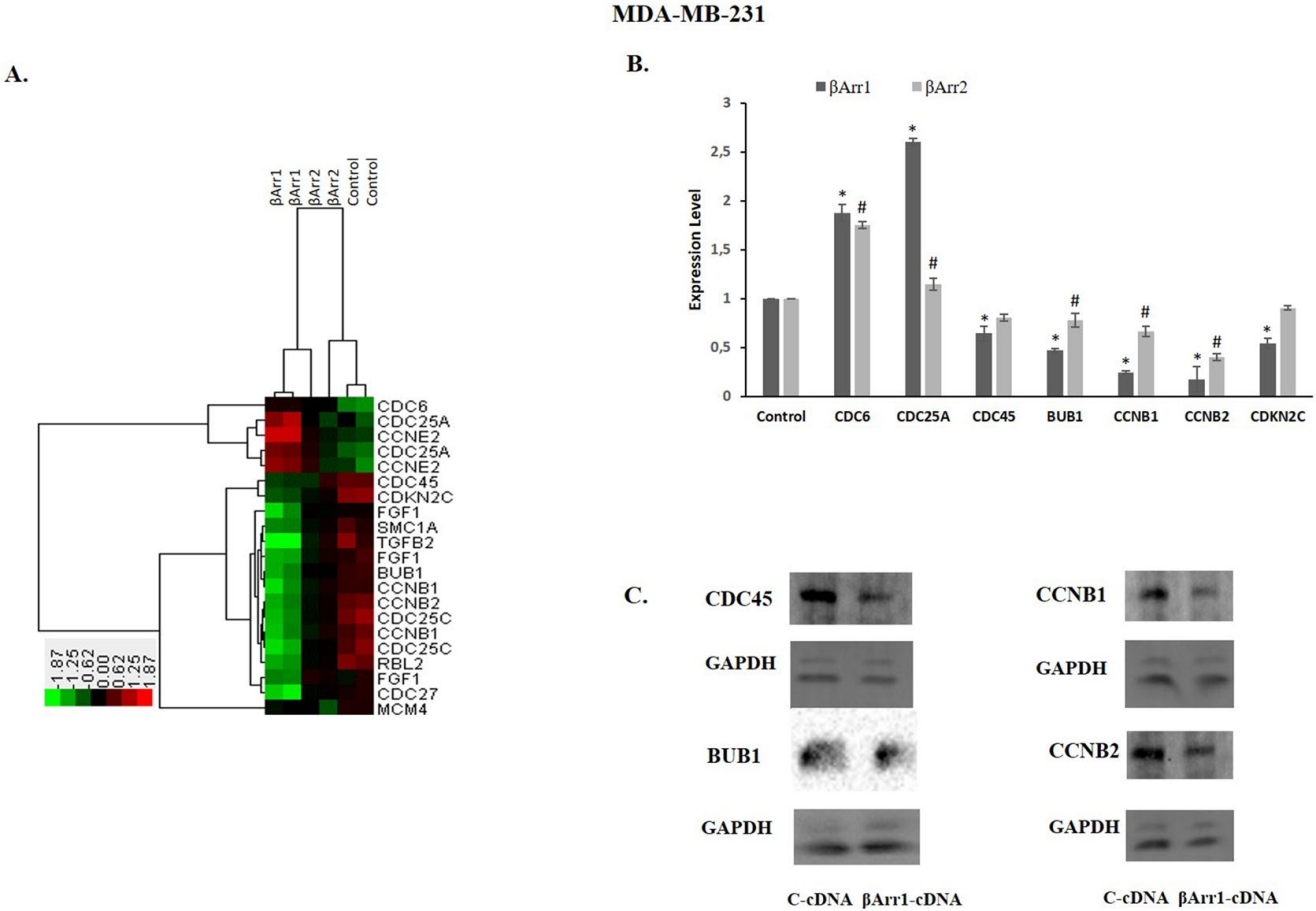
βArr1 or βArr2 overexpression affects cell cycle specific genes. (**A**) Two-way hierarchical clustering analysis was performed for the grouping of samples according to both genes (right panel) and sample types (top panel). The heatmap was visualized by Treeview. The expression profiles of the cell cycle related genes obtained from microarray analysis successfully grouped the βArr1-, and βArr2-overexpressing cells and the control cells when hierarchical clustering was performed. Green boxes represent downregulation, red boxes are used for upregulation and black means no change in the gene expression levels and the expression levels vary between − 1.87 and 1.87 folds in log2 scale. Hierarchical clustering analysis demonstrated that the expression profiles of CDC6, CDC25A, and CCNE2 were upregulated following βArr1 overexpression, and some of the genes like CDC45, BUB1, CCNB1, and CCNB2 were downregulated in those cells. (**B**) The expression profiles of the cell cycle genes were validated by qRT-PCR experiments. The expression profiles of CDC6, CDC25A were upregulated following βArr1 or βArr2 overexpression, CDC45, BUB1, CCNB1, CCNB2 and CDKN2C were downregulated in those cells. Data are presented as mean ± standard error of the mean (*p < 0.05 for βArr1; #*p* < 0.05 for βArr2 versus C-cDNA). C) The expression of some of the cell cycle genes was also validated in protein level by Western-blot analysis and expression of CDC45, BUB1, CCNB1 and CCNB2 was downregulated in βArr1 overexpressed MDA-MB-231 cell compared to control cells.

The pathway enrichment analysis showed that the cell cycle was the most significant pathway, which led us to further investigate the members of this pathway (Fig. 6). Hierarchical clustering analysis demonstrated that the expression profiles of some cell cycle signalling pathway members enabled βArr1-overexpressed cells to be successfully distinguished from control cells (Fig. 6A). While genes such as CDC6, CDC25A, and CCNE2 were upregulated following βArr1 overexpression, CDC45, BUB1, CCNB1, and CCNB2 were downregulated in those cells (Fig. 6A). Based on these results, we conducted validation experiments using qRT-PCR with cell cycle pathway members in MDA-MB-231 cells transfected with βArr1-cDNA, βArr2-cDNA, and control-cDNA. Concordant with the microarray analysis results, CDC6 and CDC25A were upregulated whereas CDC45, BUB1, CCNB1, CCNB2 and CDKN2C were downregulated in βArr1 or βArr2 overexpressed cells compared to control cells (Fig. 6B). Western-blot analysis also validated the downregulation of CDC45, BUB1, CCNB1, CCNB2 in βArr1 overexpressed MDA-MB-231 cells compared to control cells (Fig. 6C).

**Figure 7:**
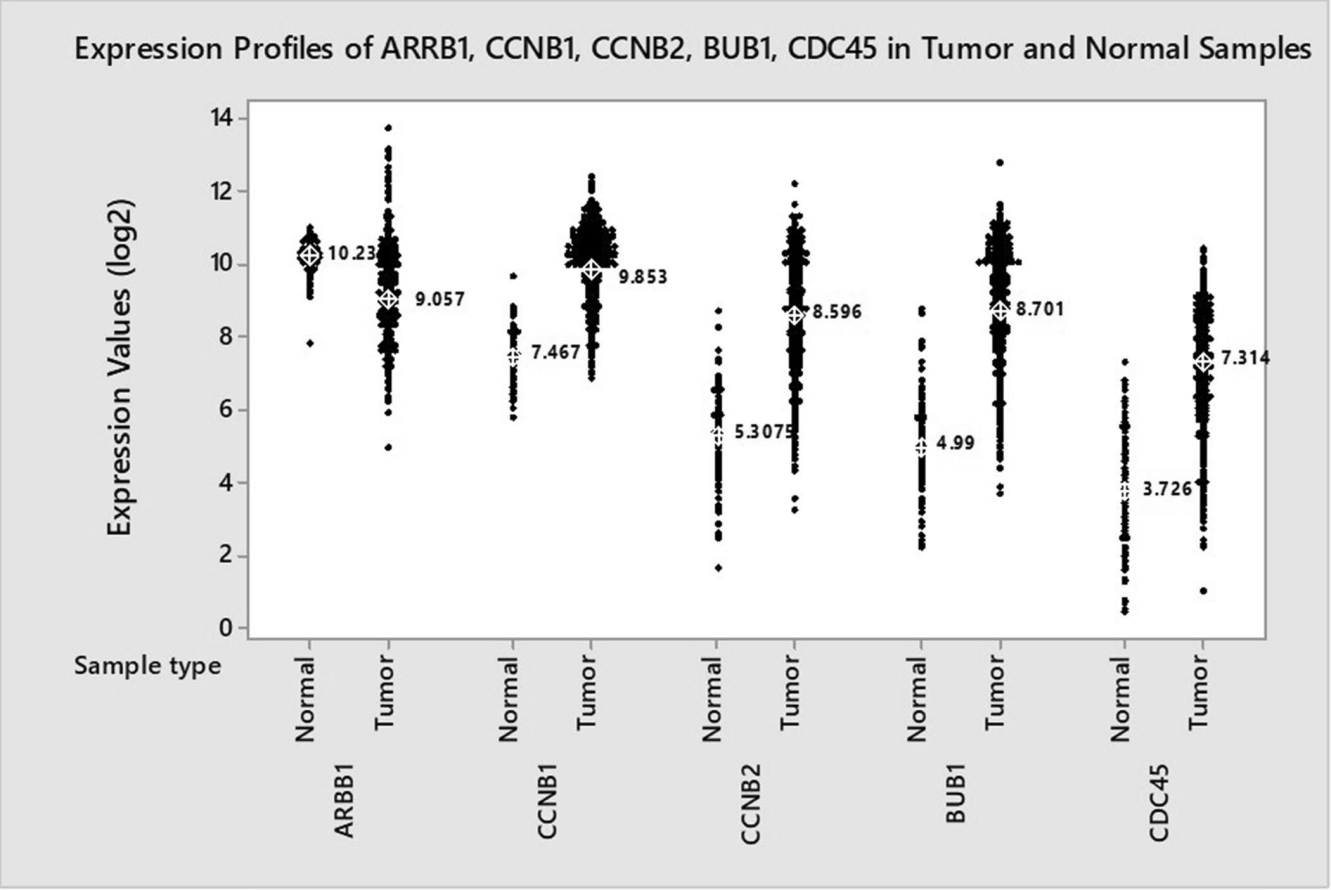
TGCA data analysis and expression profile of βArr1, CDC45, BUB1, CCNB1, CCNB2. The expression profiles of the genes in 114 normal samples and 1097 primary tumour samples obtained from TCGA are shown in individual value plots. The median expression values are marked for each sample type on the graph. When the expression profiles of βArr1(ARRB1) and cell cycle genes were evaluated in tumour and normal samples obtained from the TCGA data set, ARRB1 expression was found to be significantly downregulated in tumour samples compared to normal samples (t-test; *p* = 1.35977E^−33^). Contrary to ARRB1 expression, all the selected cell cycle genes were found to be significantly upregulated in tumour samples compared to normal ones (t-test; *p* = 6.9264E^−115^ for CCNB1; *p* = 7.7329E^−123^ for CCNB2; *p* = 9.0676E^−129^ for BUB1 and *p* = 1.3117E^−106^ for CDC45) and reversely correlated with βArr1 expression and this reverse correlation was statistically significant (Pearson correlation, r_ARRB1 vs CDC45_ = − 0.356, r_ARRB1 vs BUB1_ = − 0.394, r_ARRB1 vs CCNB1_ = − 0.388, r_ARRB1 vs CCNB2_ = − 0.397; *p* < 0.05).

Additionally, in order to examine the clinical relevance of our findings indicating the relation between the expression of these genes and βArr1 we performed TCGA data analysis and found out that CDC45, BUB1, CCNB1, and CCNB2 genes were consistently and significantly upregulated (*p* < 0.05) and reversely correlated with βArr1 expression in breast tumours compared to normal tissue samples (Fig. 7) and this reverse correlation was significant (Pearson correlation, r_ARRB1 vs CDC45_ = − 0.356, r_ARRB1 vs BUB1_ = − 0.394, r_ARRB1 vs CCNB1_ = − 0.388, r_ARRB1 vs CCNB2_ = − 0.397; *p* < 0.05).

### Regulation of βArr1 or βArr2 expression levels modulates cell motility and invasion in MDA-MB-231 and MDA-MB-468 cells

**Figure 8:**
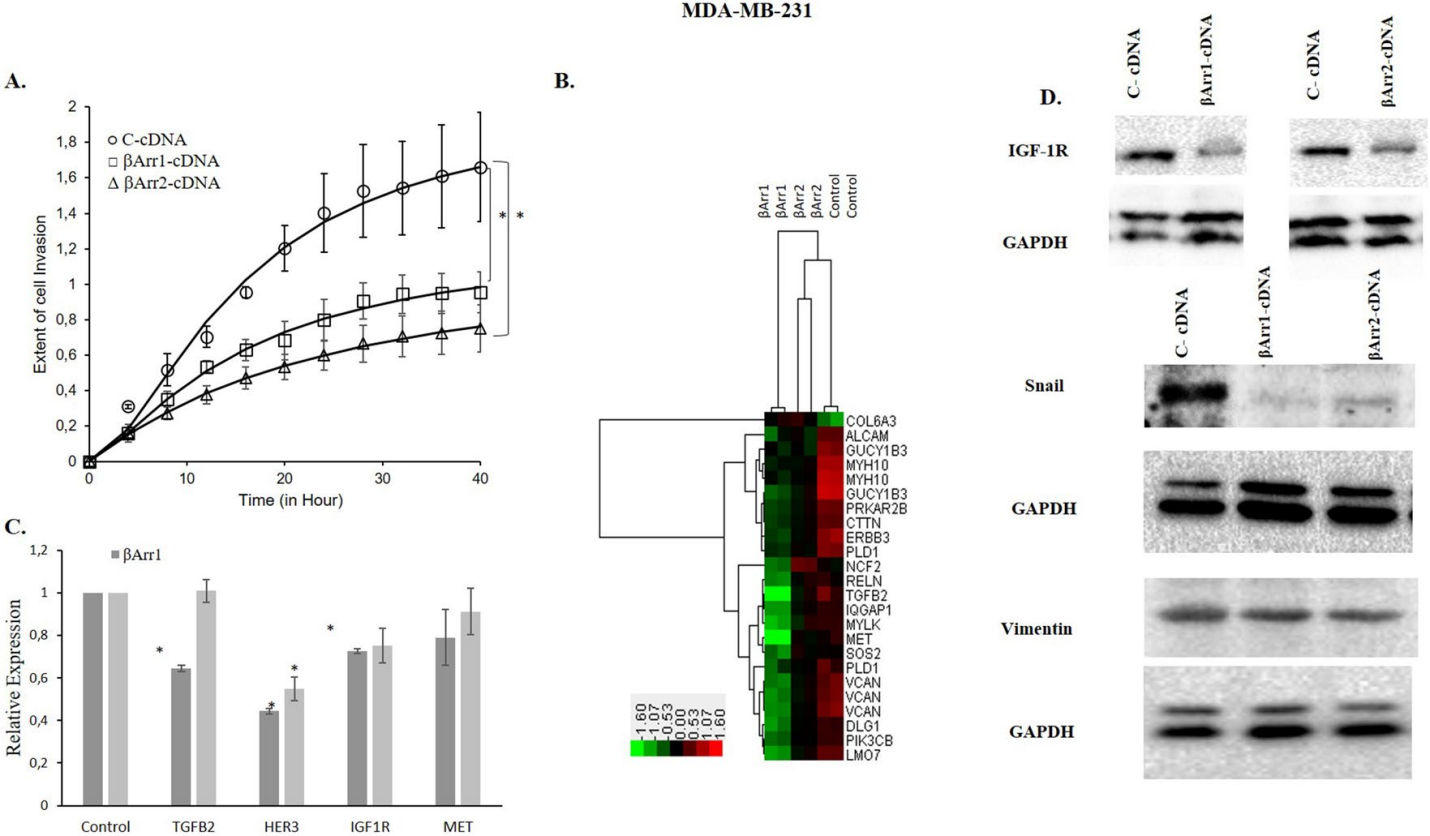
Overexpression of βArr1 or βArr2 and decreased cell invasion in MDA-MB-231. Invasion was significantly reduced in βArr1- or βArr2-cDNA transfected MDA-MB-231 cells. (**A**) Invasion of C (control-cDNA)-, βArr1- or βArr2-cDNA transfected MDA-MB-231 was monitored continuously using an xCelligence Real-Time Cell Analyzer in Matrigel coated CIM-plates. The cells were seeded in the upper chamber of the plates and impedance measurements of cell invasion were recorded at 40 h. Y axis shows changes in impedance as a measurement of the extent of cell invasion. Data are presented as mean ± standard error of the mean; n = 4–5; ^*^ = *p* < 0.05 versus C-cDNA. (**B**) Two-way hierarchical clustering analysis was performed for the clustering of samples according to both genes (right panel) and sample types (top panel). The heatmap was visualized by Treeview. Hierarchical clustering performed with the motility and invasion related genes obtained from the microarray analysis successfully separated βArr1 and βArr2 overexpressed cells from control cells. Green boxes represent downregulation, red boxes are used for upregulation and black means no change in the gene expression levels and the expression levels vary between − 1.6 and 1.6 folds in log2 scale. (**C**) The expression profiles of selected motility and invasion related genes were determined by qRT-PCR experiments and expression of TGFβ2, HER3, IGF1R was significantly down regulated. Data are presented as mean ± standard error of the mean (*p < 0.05 versus C-cDNA). (**D**) The protein expression levels of mesenchymal markers Snail, Vimentin and IGF-1R were detected by Western blot analysis. IGF-1R and Snail were downregulated in βArr1- or βArr2 overexpressed MDA-MB-231 cells while Vimentin expression stayed stable.

**Figure 9:**
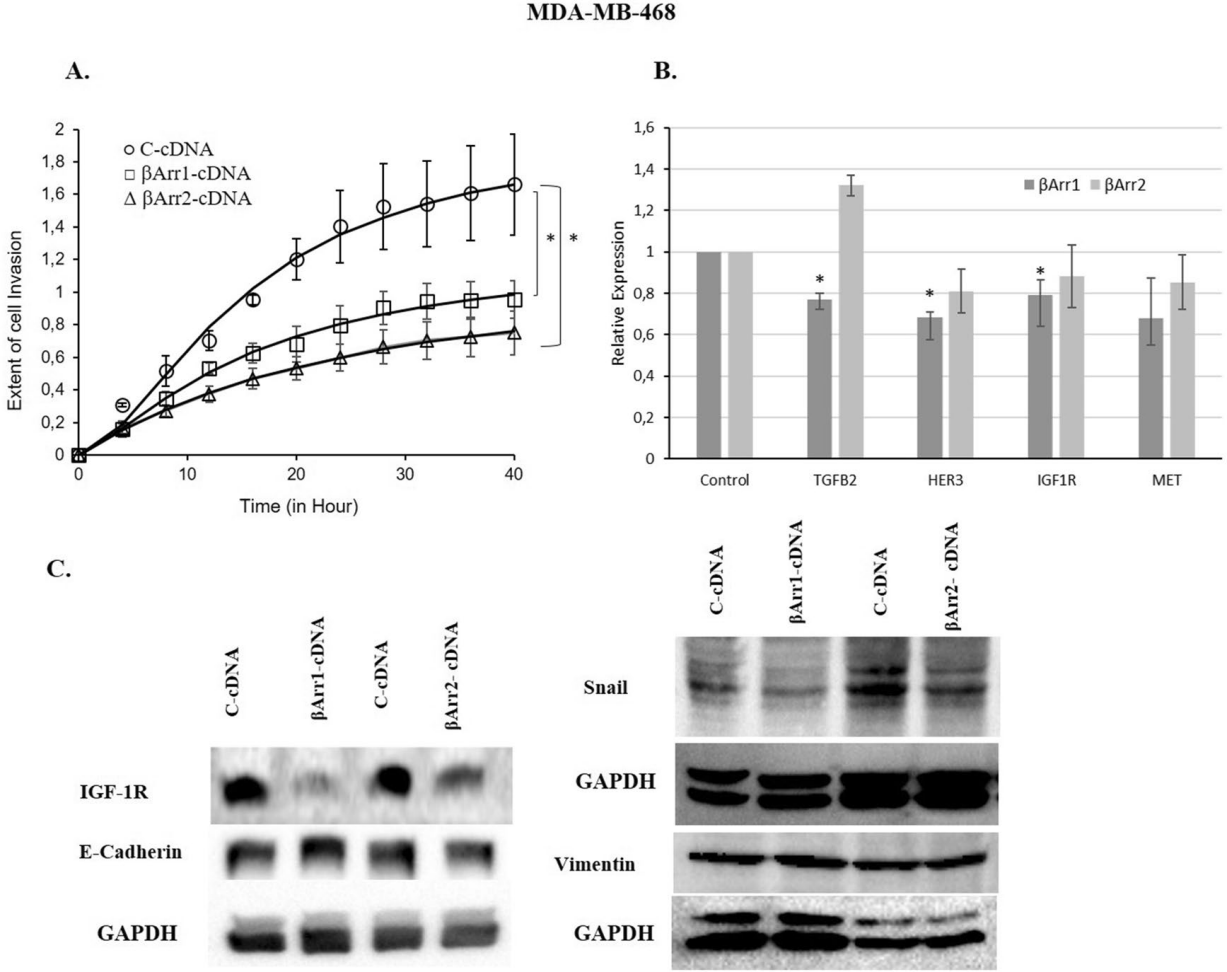
Overexpression of βArr1 or βArr2 and decreased cell invasion in MDA-MB-468. The invasion was significantly reduced in βArr1- or βArr2-cDNA transfected MDA-MB-468 cells. (**A**) Invasion of C (control-cDNA)-, βArr1- or βArr2-cDNA transfected MDA-MB-468 cells was monitored continuously using an xCelligence Real-Time Cell Analyzer in Matrigel coated CIM-plates. The cells were seeded in the upper chamber of the plates and cell invasion dependent changes in impedance were recorded at 40 h. Y axis shows changes in impedance as a measurement of extent of cell invasion. Data are presented as mean ± standard error of the mean; n = 4–5; ^*^ = *p* < 0.05 versus C-cDNA. (**B**) The expression profiles of selected motility and invasion related genes were determined by qRT-PCR experiments and expression of TGFβ2, HER3, IGF1R was significantly downregulated in βArr1-cDNA transfected MDA-MB-468 cells. Data are presented as mean ± standard error of the mean (*p < 0.05 versus C-cDNA). (**C**) In MDA-MB-468, transfection of βArr1 or βArr2-cDNA significantly increased their expression in protein levels, decreased IGF-1R and Snail expression but did not change Cadherin and Vimentin expression.

Real-time impedance-based measurements showed that overexpression of βArr1or βArr2 significantly decreased migration (data not shown) and invasion of MDA-MB-231 and MDA-MB-468 cells (Figs. 8A, 9A). Conversely, when expression of βArr1or βArr2 was downregulated by siRNAs, the cells’ invasion capacities increased significantly in both cell lines (Supplementary Fig. 2). The hierarchical clustering analysis performed with the invasion-related genes obtained from our microarray data clustered the βArr1 overexpressed MDA-MB-231 cells apart from the control cells (Fig. 8B). This analysis showed that most of the invasion related genes such as TGFβ2, HER3, MET, VCAN and PIK3CB were downregulated in βArr1 overexpressing MDA-MB-231 cells (Fig. 8B).

qRT-PCR experiments were performed with both βArr1 and βArr2 overexpressed MDA-MB-231 and MDA-MB-468 cells for the validation of microarray results. We found that TGFβ2 and IGF-1R were significantly downregulated in βArr1 overexpressed MDA-MB-231 cells while HER3 downregulation was significant following the overexpression of both βArr1 and βArr2 in these cells (Fig. 8C). These genes were also analysed in MDA-MB-468 cells, showing significant downregulation in βArr1 transfected cells (Fig. 9B).

Western blotting results confirmed the loss of mesenchymal phenotype by diminishing the protein levels of mesenchymal markers, Snail, and IGF-1R in both βArr1 or βArr2 overexpressed cell lines (Figs. 8D, 9C). On the other hand, Vimentin expression was stable in both cell lines together with Cadherin expression in MDA-MB-468 cells (Figs. 8D, 9C and Supplementary Fig. 3).

## Discussion

There is growing evidence that βArrs have a regulatory role in cancer development and progression. In general, due to the important role of βArrs in GPCR signalling, studies examining their role in cancer are mostly related to the regulation of GPCR signalling. Therefore, the significance of βArrs in cancer research come from the studies examining the influence of GPCR signalling in cancer cell behaviour. Given the diversity of different GPCR signals in cancer cell regulation, contradictory results are inevitable regarding the role of βArrs in cancer research. In this study, rather than determining their importance in cancer cell behaviour through GPCR signalling, we examined their direct influence on cellular function and gene expression profile by changing their expression levels in breast cancer cells.

First, we assessed the expression profiles of βArr1 and βArr2 in the TCGA data set to evaluate their clinical significance in breast cancers. Analysing the TCGA data showed the gradual downregulation of βArr1 from Luminal A to the most severe subtype of breast cancer, Basal-like. Tumour normal comparisons together with PAM50 classification^34^ results using TCGA data revealed the potential tumour suppressor function of this gene and underlined the prognostic value of this gene in breast cancer. In line with this result, it has also been reported that expression of βArr1 decreased with increasing breast cancer aggressiveness and was associated with positive node status, increased tumour size, nuclear grade, and decreased survival^21^. These results indicate that βArr1 is a potential prognostic marker in breast cancer. According to our TCGA analysis, despite a slight increase in the expression of βArr2 in tumour samples compared to normal samples, a significant downregulation was observed in molecular subtypes of breast cancers (Luminal A, Luminal B or Her2-enriched) compared to the Normal-like phenotype. When these results were evaluated in detail, the reverse correlation between the expression of these two genes was noticeable. βArr1 is observed to be downregulated in tumour samples while βArr2 is upregulated. Concordantly, when βArr1 expression gradually decreased from Luminal-A to Basal-like tumours conversely the βArr2 expression gradually increased from Luminal A to Basal-like subtypes. The reverse correlation between the expression levels of βArr1 and βArr2 in clinical samples may be related to the compensatory expression of these two genes^11^.

Additional to the evaluation of TCGA data, the expression of βArr1or βArr2 in breast cancer cell lines was determined. We found that expression of βArr1or βArr2 was significantly less in the most aggressive breast cancer cell lines (TNBC cells MDA-MB-231 or MDA-MB-468) than less aggressive ones (SKBR3 or BT4749). These findings are in agreement with the results of a previous study^21^. Furthermore, the expression level of these was lowest in MDA-MB-231 which is the most aggressive and invasive compared to MDA-MB-468 and the others^39^.

Based on these results, we investigated how altering the expression level of βArrs affects the behaviour of TNBC cells. Downregulation of βArr1 or βArr2 increased proliferation and facilitated the migration and invasion of both MDA-MB-231 and MDA-MB-468 cells. The effect of downregulation on the proliferation was more pronounced in MDA-MB-468 cells, which may be related to the higher expression level of βArrs in these cells compared to MDA-MB-231 cells. Conversely, upregulation of βArr1 or βArr2 reduced proliferation, migration, and invasion of both MDA-MB-231 and MDA-MB-468 cells. This time, the effect of up-regulation on the proliferation was more remarkable in MDA-MB-231 cells, which also might be related to the lower expression level of βArrs in these cells. These results support the potential tumour suppressor role of these proteins in TNBC cells. Despite the presence of contradictory results which highlight the diverse and complex influences of βArrs in regulating cell proliferation, apoptosis, invasion, tumour development, etc.^5,6,13^, some studies are in line with our results. The proliferation and invasion abilities of renal cell carcinoma cell lines increased when the expression level of βArr2 was decreased^17^. Down regulation of βArr2 increases hepatocellular carcinoma cell migration and invasion while its low expression level may be related to a poor prognosis^16^. Depletion of βArr2 promoted angiogenesis, tumour development, and metastasis in two different murine lung cancer models^19^.

To determine the link between βArrs and the molecular mechanisms of their potential anticancer response pattern in TNBC cells, we performed microarray analysis. This revealed differentially expressed genes in βArr1 or βArr2 overexpressing MDA-MB-231 cells. The hierarchical clustering and pathway enrichment analyses performed with the differentially expressed genes showed the significant influence of the overexpression of these proteins on cell-cycle, p53, and JAK-STAT signalling pathways particularly.

Based on these results showing that overexpression of βArr1 or βArr2 influences cell cycle genes and reduces cell proliferation, we evaluated the cell cycle in βArr1- and βArr2-overexpressed MDA-MB-231 and MDA-MB-468 cells. We found that increasing the expression level of βArr1 or βArr2 prolonged the cell cycle in the S phase. We believe this result can explain the decline in cell proliferation in TNBC cells.

The microarray results validated by the qRT-PCR experiments on the cell cycle pathway genes showed that CDC6 and CDC25A were upregulated in βArr1 and βArr2 overexpressed cells compared to the control cells. CDC6 is a key protein for DNA replication and there is evidence that CDC6 upregulation prevents mitosis^40^. This can be related to the βArr1 or βArr2 overexpression mediated antiproliferative response observed in this study. The activity of cyclin dependent kinases (CDKs), which regulate cell cycle progression, is closely controlled by dephosphorylating enzyme CDC25A^41^. Excessive or abnormal CDK activity leads to DNA-double strand breakage and DNA damage^42,43^. Upregulation of CDC25 in βArr1 or βArr2 overexpressed cells can increase abnormal CDK activity and could be one of the underlying mechanisms of S phase prolongation. Additionally, our data showed that CDKN2C is down regulated with the overexpression of βArr1 or βArr2. Down regulation of CDKN2C (p18), which is an inhibitor of CDK4 and CDK6, can also increase abnormal CDK activity. Inactivation of CDKN2C (p18), which inhibits CDK4 and CDK6, suppresses the cellular growth of one of the TNBC cells, BT-20^44^. This supports our results that excessive and abnormal CDK activity may interfere with the cell cycle and cell proliferation in TNBCs. The other underlying mechanism for the effect of βArr1 or βArr2 overexpression on proliferation and the cell cycle may be related to down regulation of CDC45, whose presence and activity is important in proliferating cells and the S phase of the cell cycle. Its level was also higher in human cancer cells than healthy human cells^45,46^. The other important gene and related protein is BUB1, whose expression is negatively correlated with breast cancer prognosis. Its depletion reduces the cancer stem cell potential of MDA-MB-231 and has anticancer effectiveness in xenograft mice cancer models^47^. We found that its expression was significantly downregulated with the overexpression of βArr1 or βArr2 in MDA-MB231 cells in this study. The other two genes that are downregulated with overexpression of βArr1 or βArr2 are CCNB1 and CCNB2. These cancer related proteins are highly expressed in several cancer types, including breast cancer. Therefore, some studies suggest measuring their expression level as a biomarker for cancer progression^48–51^. Downregulation of these genes also inhibits tumour growth and enhances the effectiveness of anticancer drugs^52,53^.

TCGA data analysis was performed for the clinical significance of our microarray analysis results showing a reverse relation between the expression of βArr1 and some of the cell cycle genes, This analysis showed that the expression level of CDC45, BUB1, CCNB1, and CCNB2 genes was consistently upregulated and that of βArr1 was downregulated in breast tumours compared to normal tissue samples. This reverse correlation obtained from TCGA analysis supports the clinical relevance of our results.

There are some contradictory results about the influence of βArr1 or βArr2 on cell motility, migration, and invasion^5,13,16,19,23^. Several studies report that GPCR mediated cell motility depends on βArrs while suppression of βArr2 expression attenuates CXCR4 receptor mediated cell migration^5,13,23,54,55^. On the other hand, the downregulation of βArr2 promoted tumour metastasis and invasion in a murine model of lung cancer and hepatocellular carcinoma, respectively^16,19^. Our results show that overexpressing βArr1 or βArr2 significantly reduces cell migration (data not shown) and invasion in MDA-MB-231 and MDA-MB-468 cells.

To better understand the underlying mechanisms of these observations, we determined differentially expressing genes and proteins that play a significant role in cell motility. We found that upregulation of βArr1 or βArr2 significantly reduces the expression of IGF-1R, HER3, and Snail. In support of this, βArr1 and βArr2 are crucial for down regulation of IGF-1R by degradation via ubiquitination^56,57^. The influence of βArr1 or βArr2 on the IGF-1R signalling pathway may also indicate their potential inhibitory effect on cell proliferation, invasion, and tumour development in TNBC cells.

HER3 is an important regulator of cancerous behaviour in breast cancer^58,59^. All the activated signalling pathways triggered by HER3 heterodimerization with HER2 or EGFR increase cancer cell proliferation, tumour growth, angiogenesis, invasion, and metastasis ^58,59^. There are several known molecular markers of epithelial-mesenchymal transition related to cell motility and metastasis. Invasiveness and metastasis are associated with low E-cadherin expression but high Vimentin and Snail expression^60^. We found that Snail declined in MDA-MB-231 cells, which did not significantly express E-cadherin. Snail, but not Vimentin, declined in MDA-MB-468 by overexpressing βArr1 or βArr2. We conclude that the influence of overexpression of βArr1 or βArr2 on the motility of these cells can be mainly related their effect on the expression level of IGF-1R, HER3 and Snail.

To the best of our knowledge, this study is the first to demonstrate the effect of βArr1 and βArr2 on the expression of cell cycle related genes, CDC45, BUB1, CCNB1, CCNB2 and CDKN2C and also of proliferation, motility and invasion related genes HER-3, and Snail. Several studies show that βArr1 and βArr2 may have regulatory effects on the expression of various nuclear genes^9,61^. βArrs can translocate to nucleus and have an influence on DNA function and transcription^6^. Overexpression of βArr1 and βArr2 might increase their translocation to nucleus and have an influence on DNA, leading a change in the expression of cell cycle genes observed in this study.

However, further studies are needed to clarify the underlying mechanisms of these changes, including the expression level of IGF-1R. Since βArr1 seems to be more clinically significant according to the TCGA data analysis and our microarray results, the molecular basis of the differences between the anticancer effect of βArr1 and βArr2 must be clarified in TNBCs.

In conclusion, increasing the expression of βArr1 and βArr2 regulates the cancer behaviour of TNBC cells by decreasing their proliferation and invasion capacity. The molecular analyses revealed that the most prominent regulatory effect of βArrs is cell cycle arrest in the S phase through their influence on the expression levels of cell cycle genes. TCGA data analysis also indicates the clinical relevance of our results, the low expression levels of βArr1 are inversely correlated with CDC45, BUB1, CCNB1, and CCNB2 genes compared to normal tissue samples while positively correlated with poorer prognosis in breast tumours. Furthermore, βArrs can have anticancer response pattern through reducing expression of growth factor receptors, IGF-1R, HER3, and the cell motility related protein, Snail. The anticancer and tumour suppressor effectiveness of βArr1 or βArr2 should thus be examined further through preclinical breast cancer models in animals.

## Supplementary Information

Supplementary Figures.

## Abbreviations

βArrs: β-Arrestins
ARRB: β-Arrestin gene
HER3: Human epidermal growth factor receptor 3
EGFR: Epidermal growth factor receptor
IGF-1R: Insulin-like growth factor receptor 1
TNBC: Triple negative breast cancer
