# Supplementary material for: Overexpression of β-Arrestins inhibits proliferation and motility in triple negative breast cancer cells
Source: Sci Rep. 2021 Jan 15;11:1539. doi: 10.1038/s41598-021-80974-6 (PMC7810837; doi:10.1038/s41598-021-80974-6)
Supplement: Supplementary file 1 — Supplementary Figures. [file 41598_2021_80974_MOESM1_ESM.pdf]

Overexpression of β-Arrestins inhibits proliferation and motility in triple negative breast cancer cells.

Saber Yari Bostanabad[1], Senem Noyan[1], Bala Gur Dedeoglu[1], Hakan Gurdal[2]

1Biotechnology Institute of Ankara University, 06135 Ankara, Turkey (SYB, SN, BGD)

2Department of Medical Pharmacology, Faculty of Medicine, University of Ankara, 06230 Ankara, Turkey (HG)

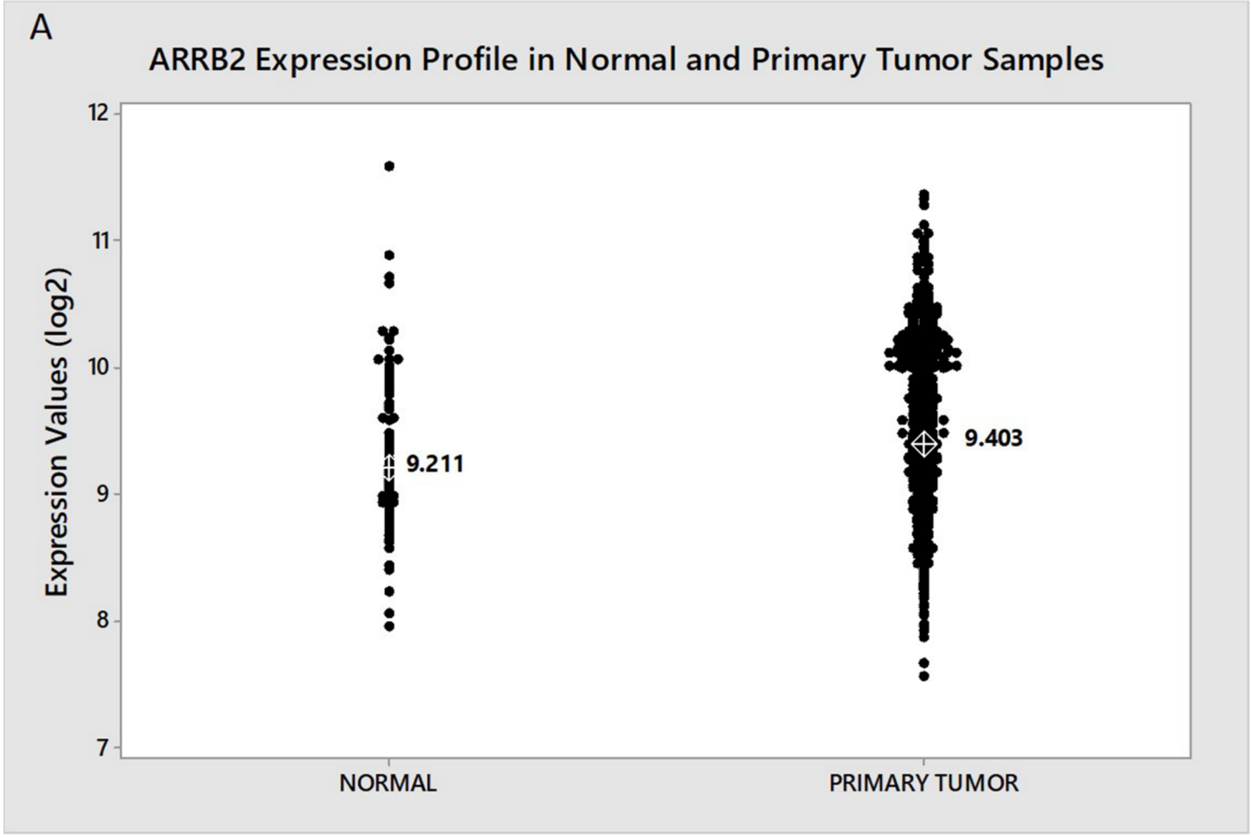

A
ARRB2 Expression Profile in Normal and Primary Tumor Samples
Expression Values (log2)
12
11
10
9
8
7
9.211
9.403
NORMAL
PRIMARY TUMOR

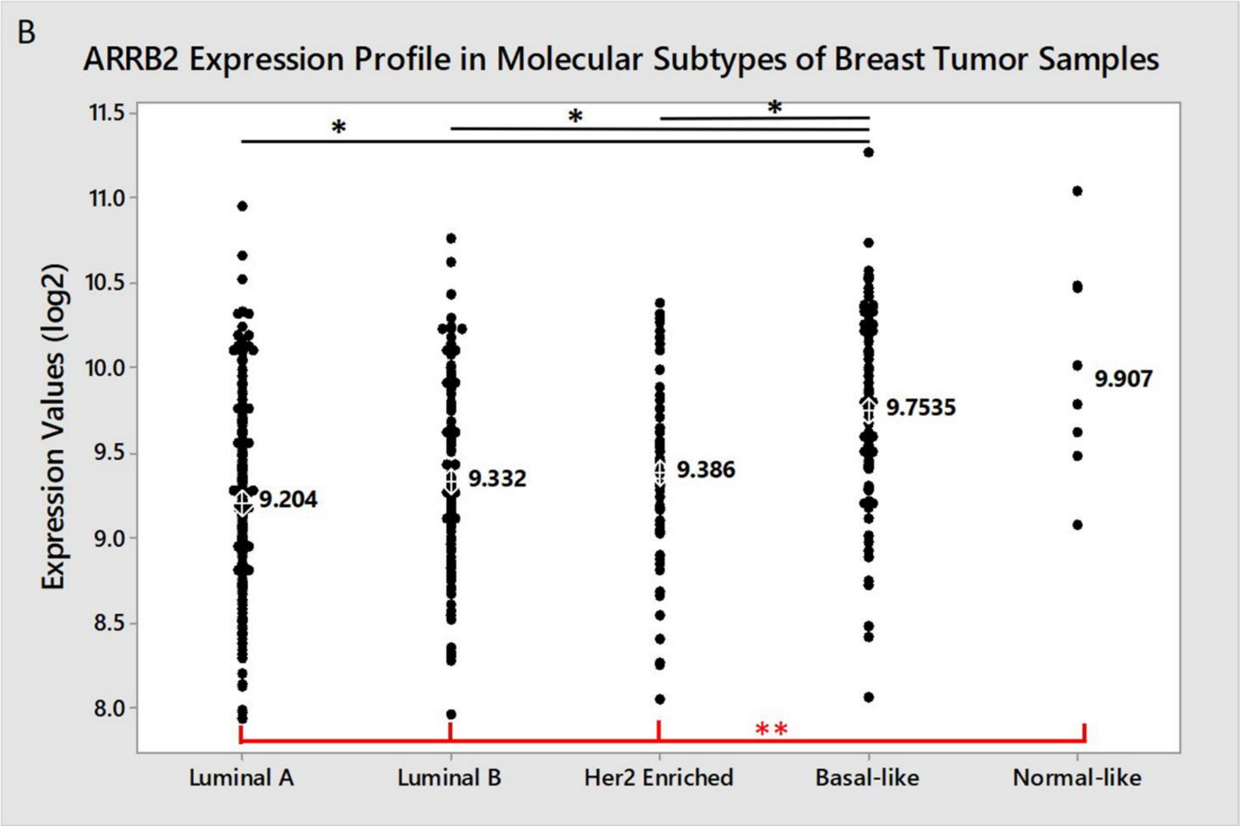

Supplementary Figure 1. TGCA data analysis results for βArr2. A) The expression profiles of βArr2 **(**ARRB2) gene in 114 normal samples and 1097 primary tumour samples obtained from TCGA are shown in individual value plots. The median expression values are marked for each sample type, which is 9.211 for normal samples and 9.403 for the tumour samples. ARRB2 gene is significantly upregulated in tumour samples compared to normal samples (t-test; p=0.027). B) The expression profiles of ARRB2 gene in 98 basal-like, 58 HER2-enriched, 127 luminal B, 231 luminal A and 8 normal-like breast tumour samples obtained from TCGA are shown in individual value plots (The median values for each sample type are given on the value plots). According to ANOVA results ARRB2 expression is downregulated significantly in Luminal A, Luminal B or Her2 Enriched compared to the Normal-like or Basal- like breast tumours (ANOVA; *p<0.0001,**p<0.005). ARRB2 expression is not significantly different between Normal-like and Basal-like breast tumours (ANOVA; p >0.05).

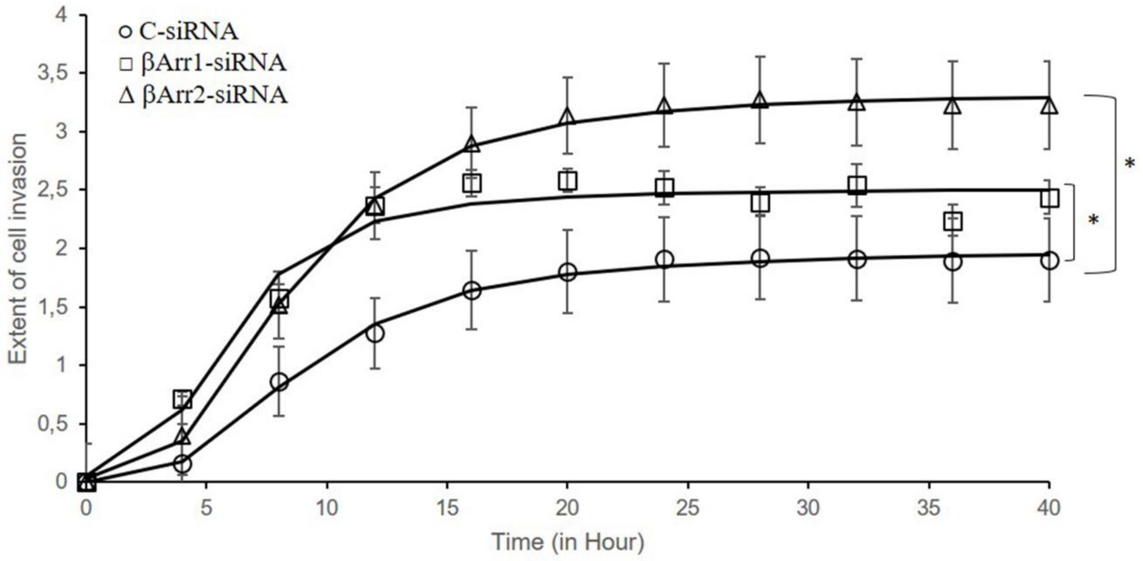

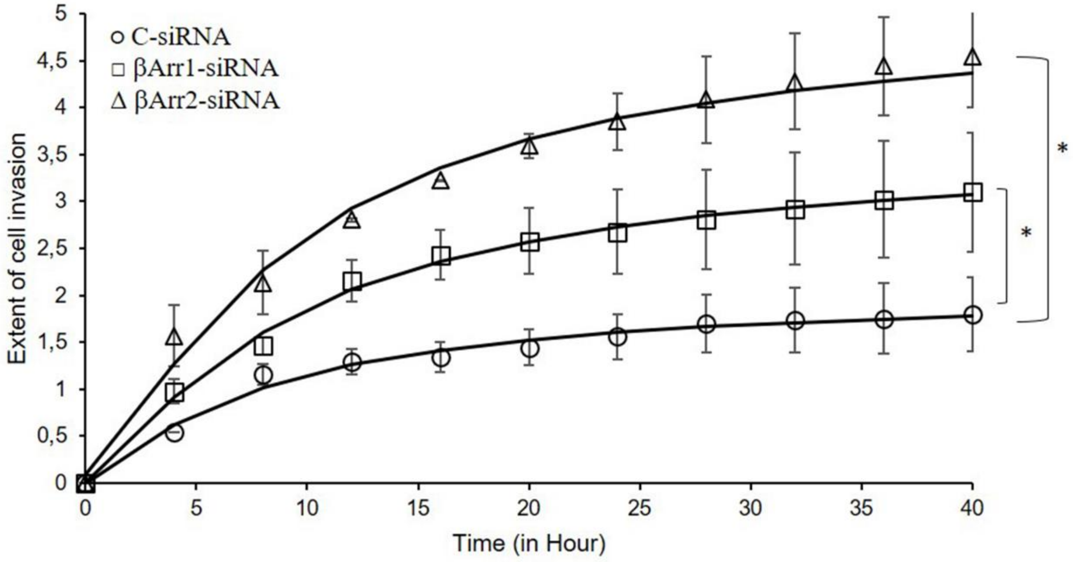

Supplementary Figure 2. Silencing expression of βArr1 or βArr2 and cell invasion.

Invasion was significantly increased in βArr1- or βArr2-siRNA transfected MDA-MB-231 and MDA-MB-468 cells. Invasion of C (control-siRNA)-, βArr1- or βArr2-siRNA transfected cells was monitored continuously using an xCelligence Real-Time Cell Analyzer in Matrigel coated CIM-plates. The cells were seeded in the upper chamber of the plates and impedance measurements of cell invasion were recorded at 40 h. Y axis shows changes in impedance as a measurement of extent of cell invasion. Data are presented as mean ± standard error of the mean; n=4-5; * = $P<0.05$ versus C-siRNA.

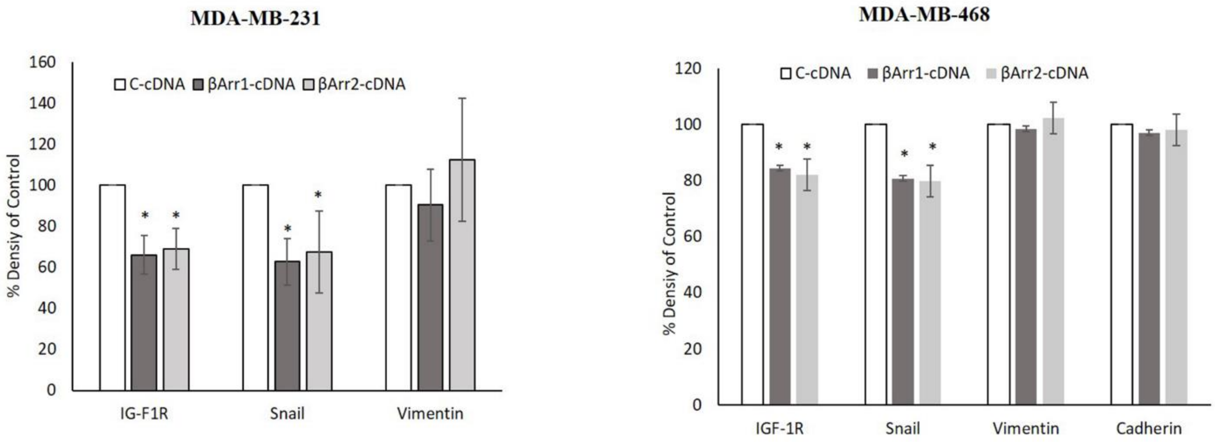

Supplementary Figure 3. In MDA-MB-231 and MDA-MB-468, transfection of βArr1 or βArr2-cDNA significantly decreased IGF-1R and Snail expression but did not change Cadherin and Vimentin expression. Bar graphs show the densitometric analysis of band intensities, normalized to GAPDH. Data are presented as mean ± standard error of the mean; n=4-5; * = P<0.05 versus C-cDNA.

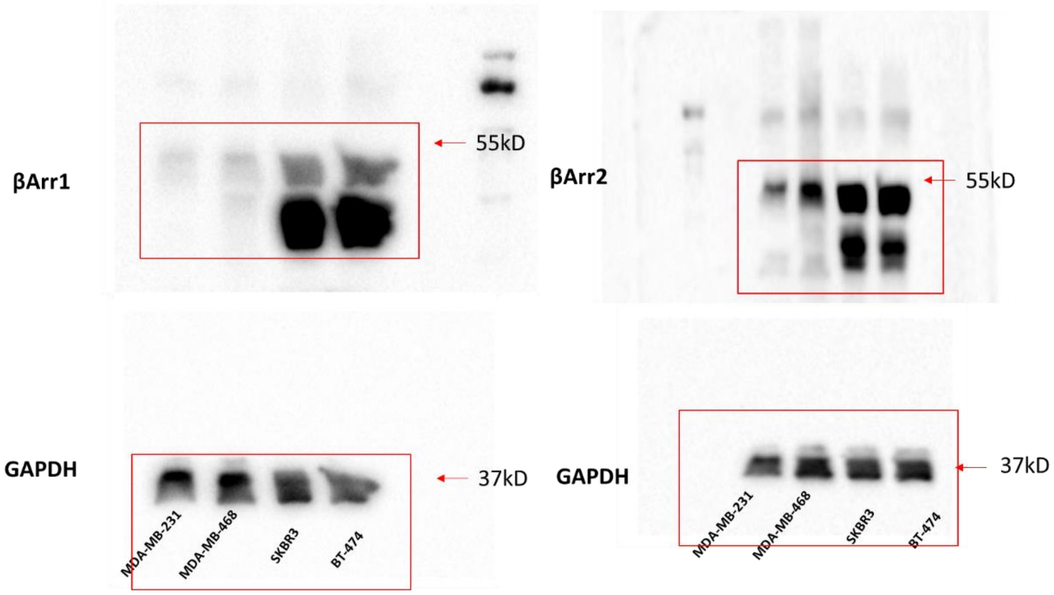

Supplementary Figure 4. The expression of βArr1 or βArr2 in the most aggressive breast cancer cell lines, TNBC cells (MDA-MB-231 and MDA-MB-468) and the less aggressive ones Her2 Enriched (SKBR3) and Luminal B (BT474). This supplementary figure is related to Figure 2.

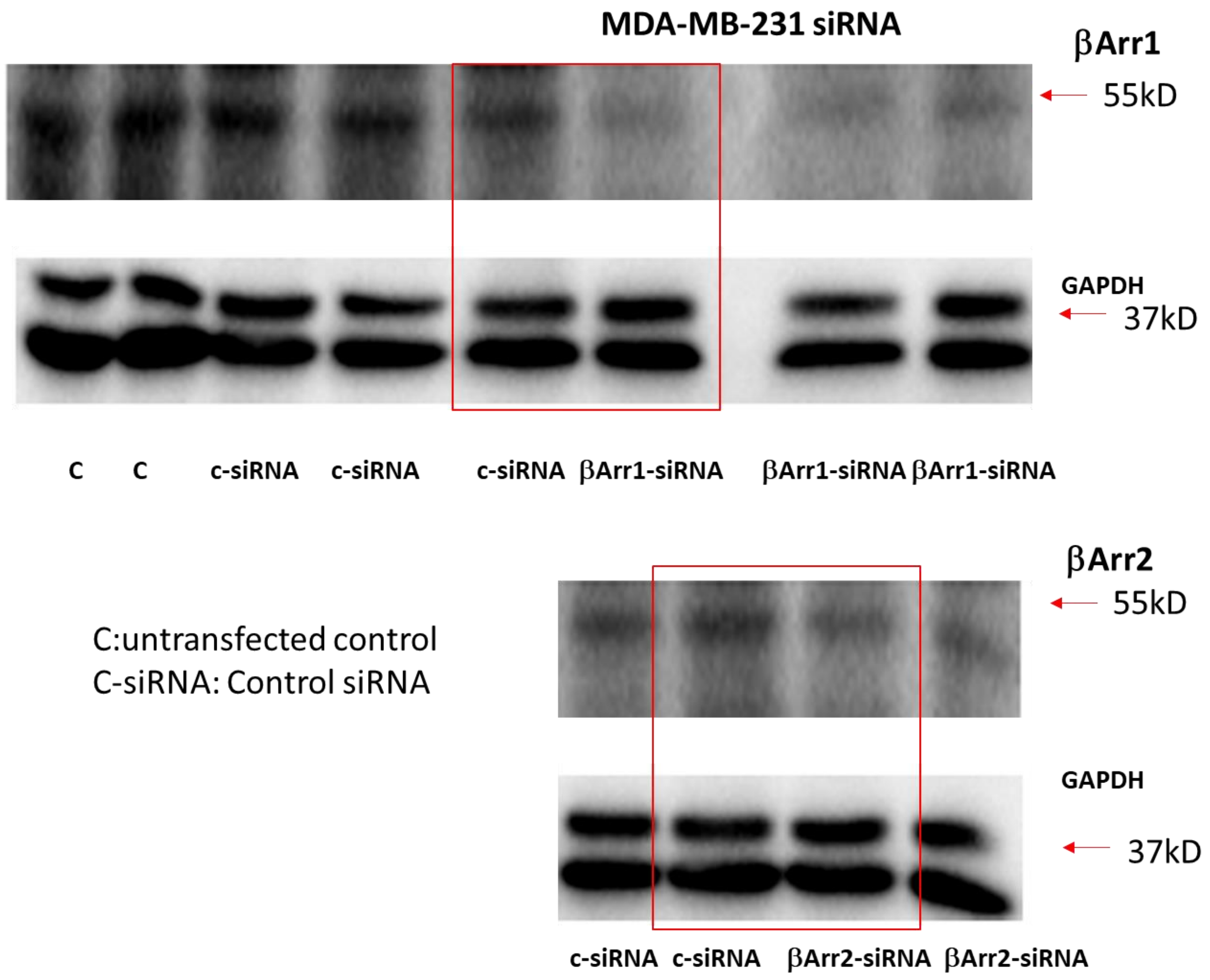

Supplementary Figure 5. The expression of βArr1 or βArr2 in control, control-siRNA, βArr1-siRNA or βArr2-siRNA transfected MDA-MB-231 cells. This supplementary figure is related to Figure 3.

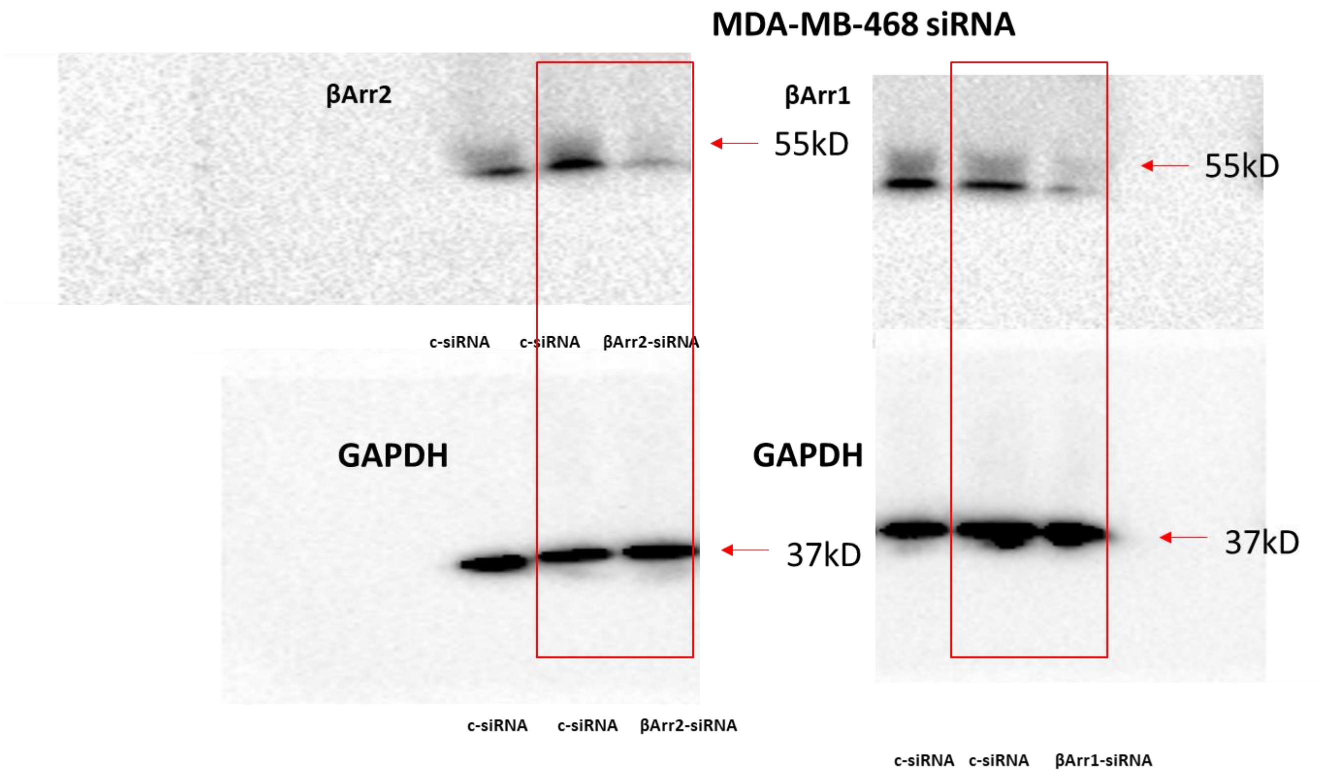

Supplementary Figure 6. The expression of βArr1 or βArr2 in control, control-siRNA, βArr1-siRNA or βArr2-siRNA transfected MDA-MB-468 cells. This supplementary figure is related to Figure 3.

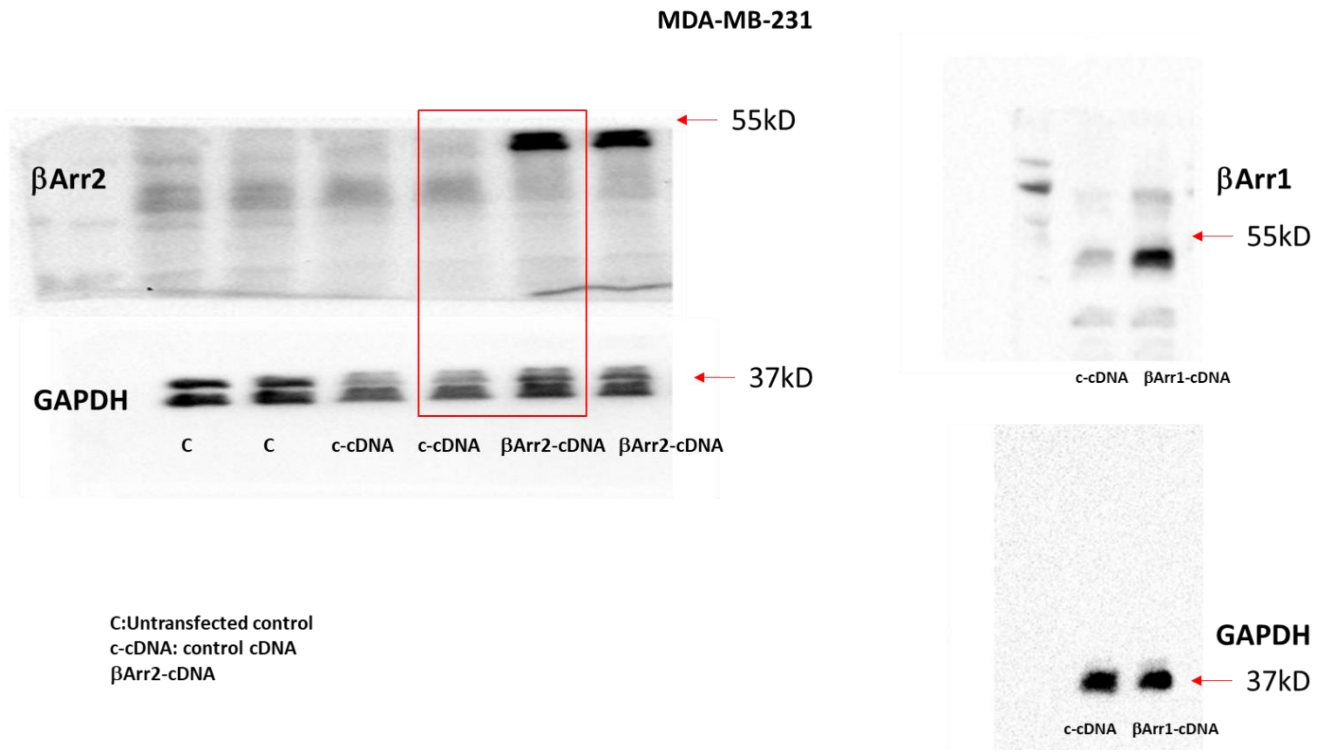

Supplementary Figure 7. The expression of βArr1 or βArr2 in control, control-cDNA, βArr1-cDNA or βArr2-cDNA transfected MDA-MB-231 cells. This supplementary figure is related to Figure 4.

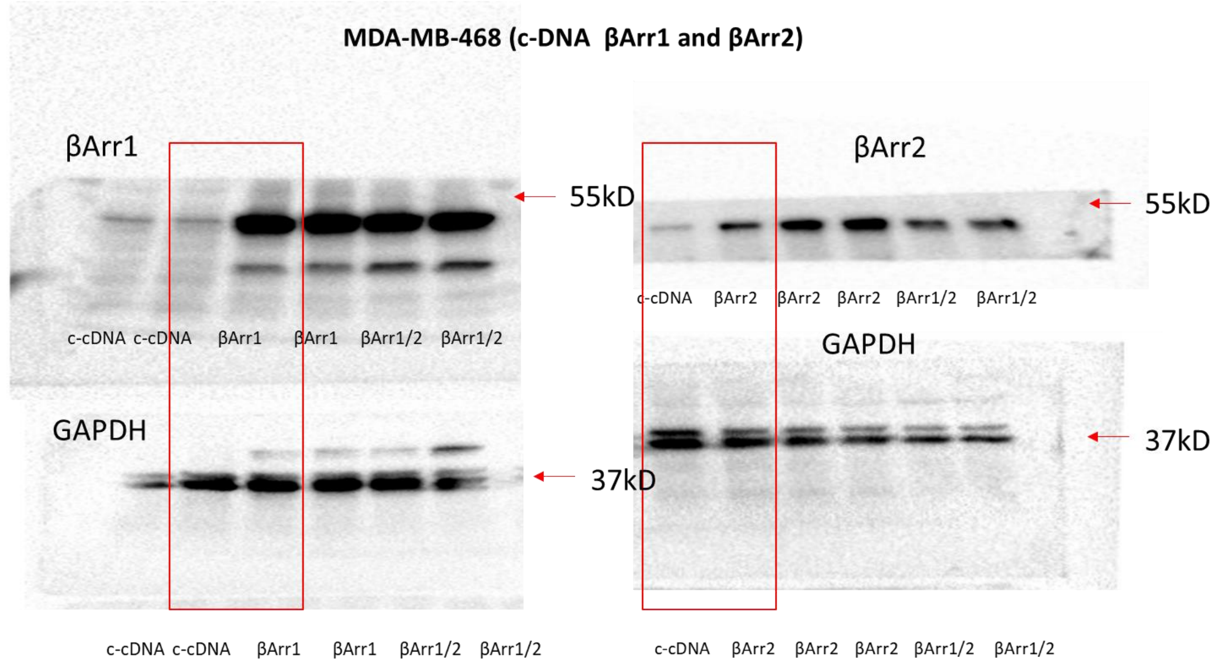

Supplementary Figure 8. The expression of βArr1 or βArr2 in control, control-cDNA, βArr1-cDNA or βArr2-cDNA transfected MDA-MB-468 cells. This supplementary figure is related to Figure 4.

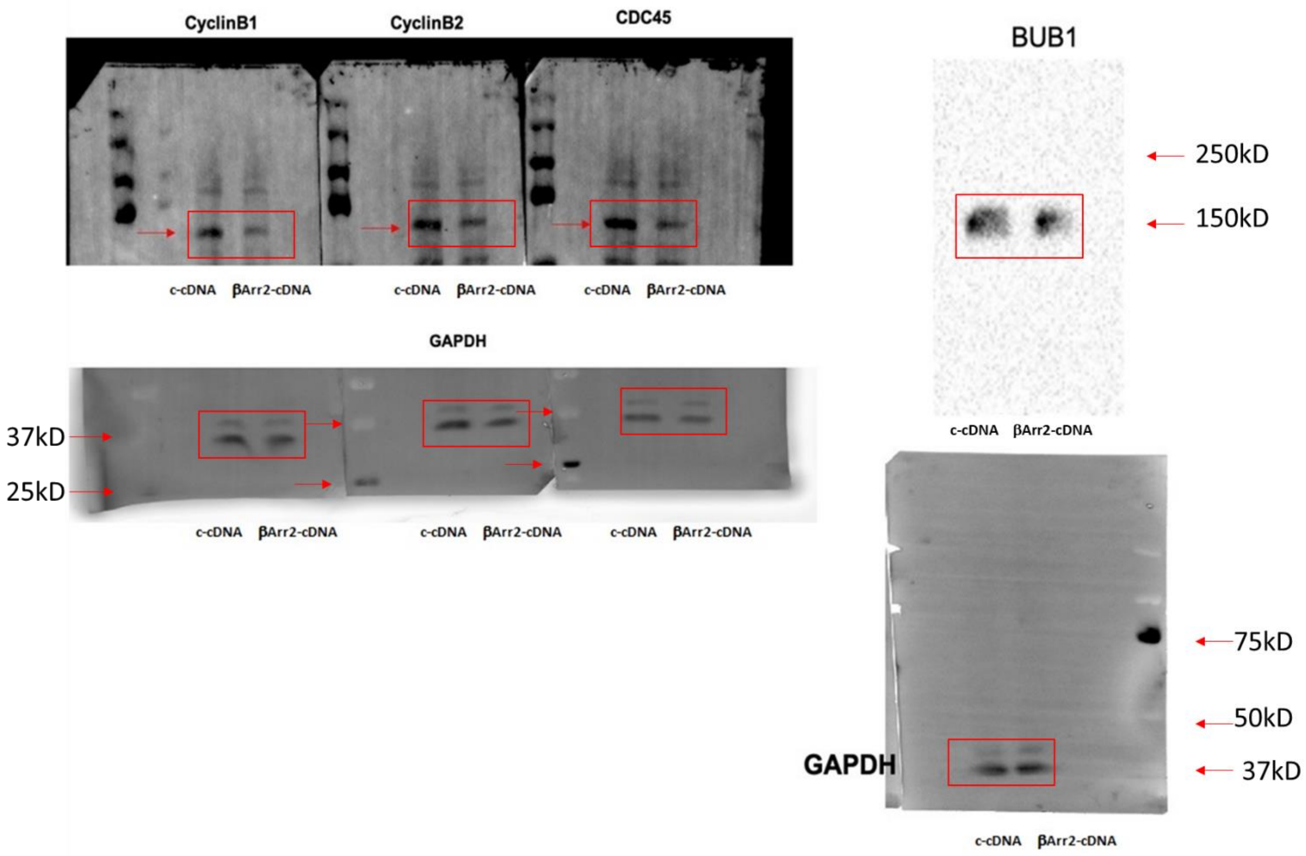

Supplementary Figure 9. The expression of CDC45, BUB1, CCNB1 and CCNB2 in control and βArr1 overexpressed MDA-MB-231 cells. This supplementary figure is related to Figure 6.

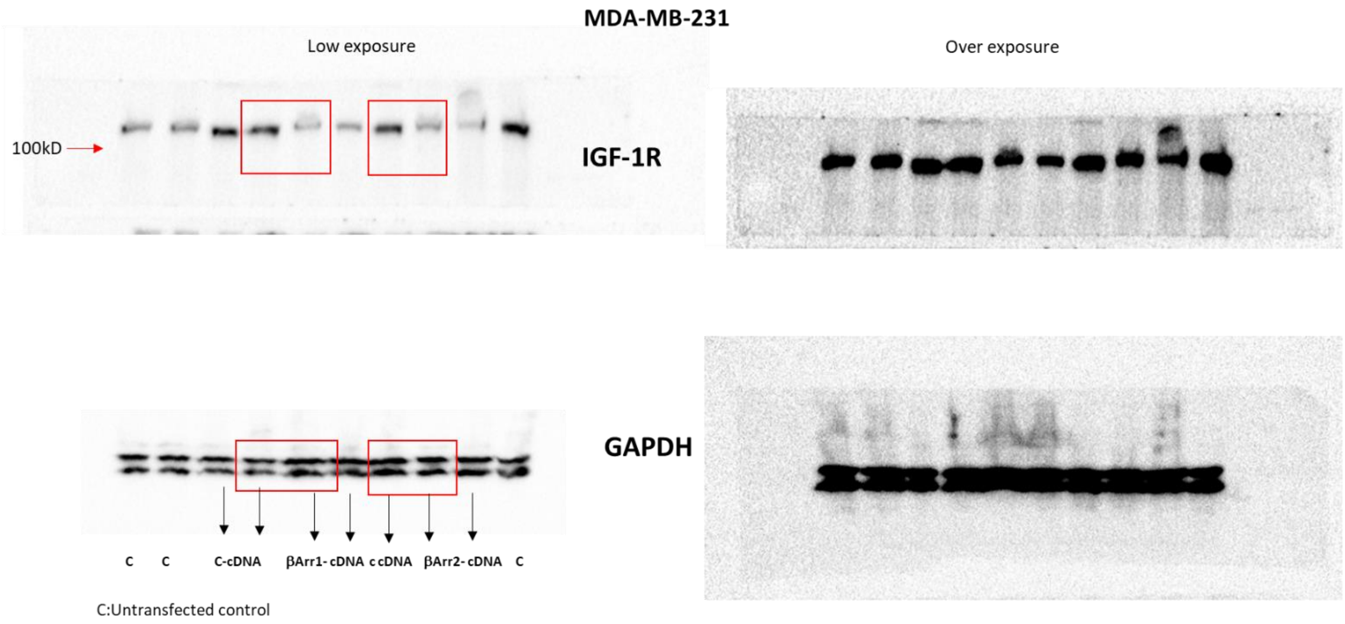

Supplementary Figure 10. The expression of IGF-1R in control (untransfected), control-cDNA, βArr1-cDNA or βArr2-cDNA transfected MDA-MB-231 cells. This supplementary figure is related to Figure 8.

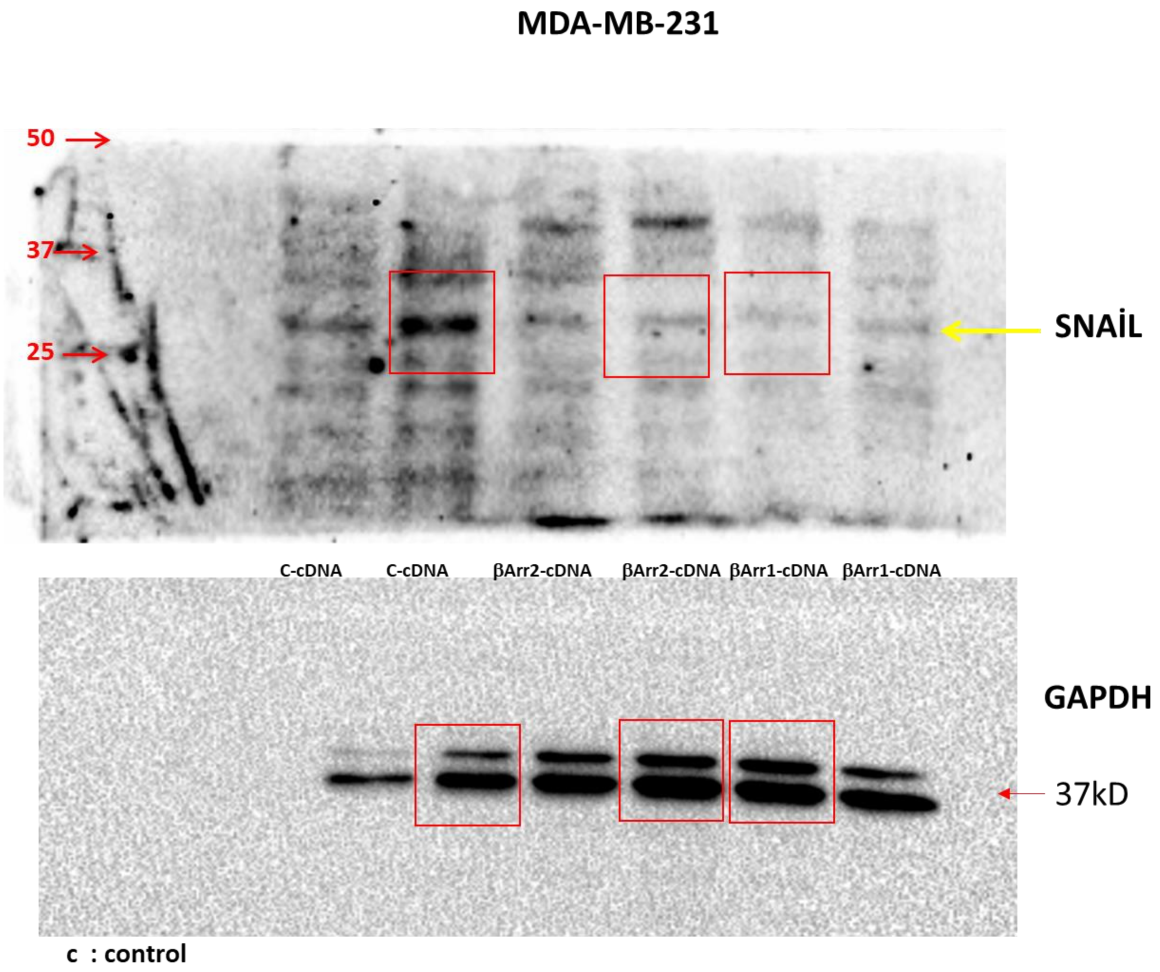

Supplementary Figure 11. The expression of Snail in control-cDNA, βArr1-cDNA or βArr2-cDNA transfected MDA-MB-231 cells. This supplementary figure is related to Figure 8.

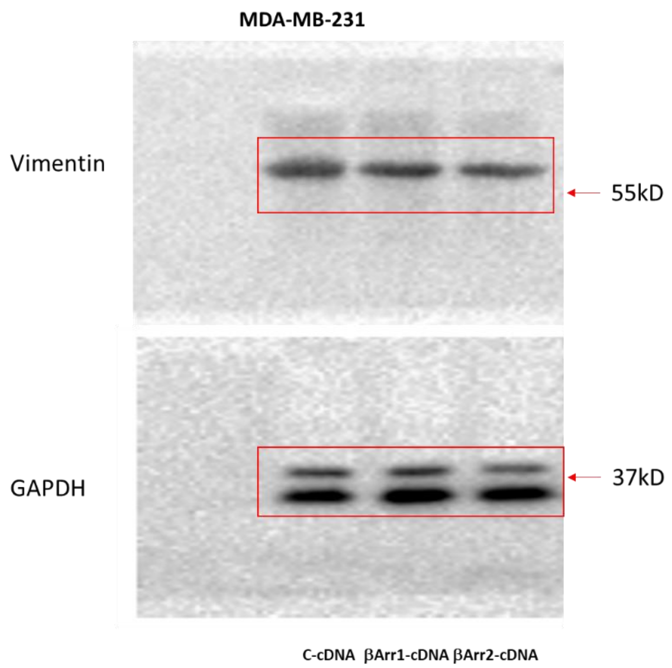

Supplementary Figure 12. The expression of vimentin in control-cDNA, βArr1-cDNA or βArr2-cDNA transfected MDA-MB-231 cells. This supplementary figure is related to Figure 8.

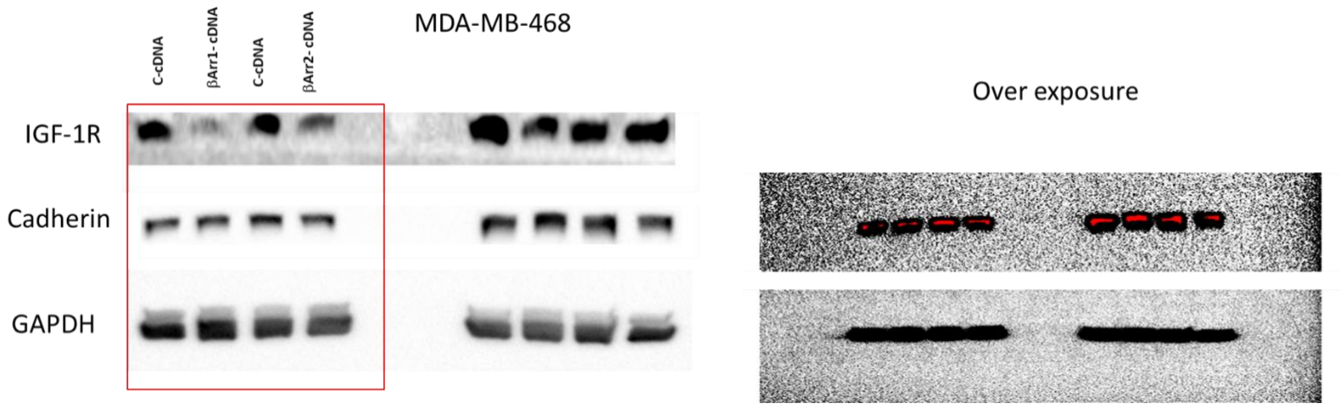

Supplementary Figure 13. The expression of IGF-1R and cadherin in control-cDNA, βArr1-cDNA or βArr2-cDNA transfected MDA-MB-468 cells. This supplementary figure is related to Figure 9.

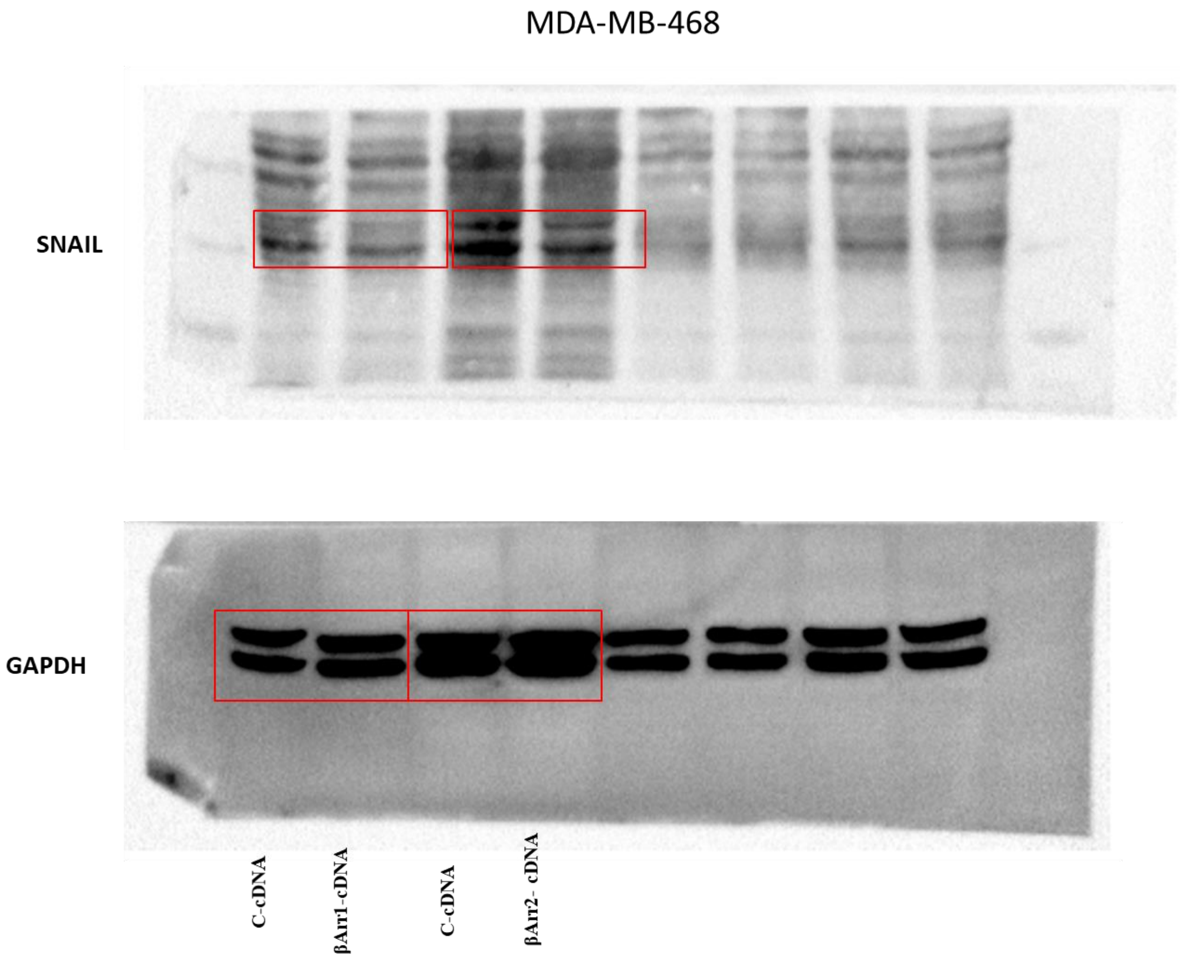

Supplementary Figure 14. The expression of snail in control-cDNA, βArr1-cDNA or βArr2-cDNA transfected MDA-MB-468 cells. This supplementary figure is related to Figure 9.

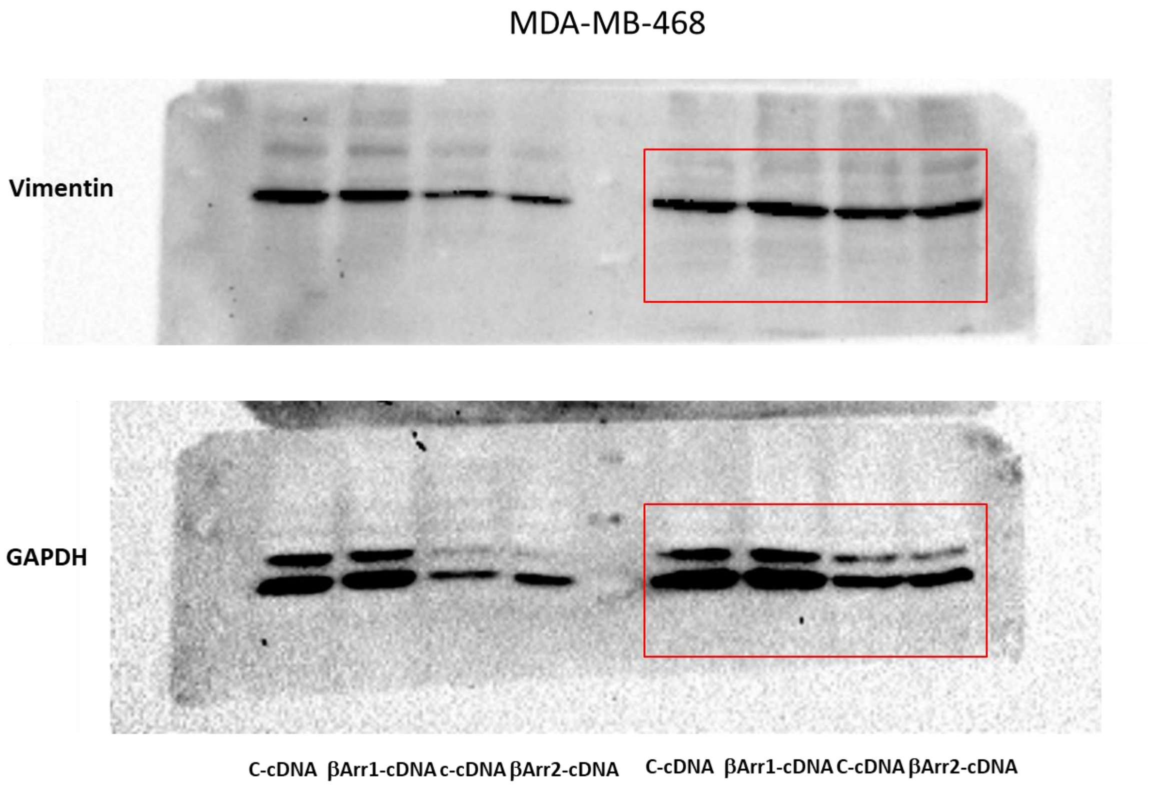

Supplementary Figure 15. The expression of vimentin in control-cDNA, βArr1-cDNA or βArr2-cDNA transfected MDA-MB-468 cells. This supplementary figure is related to Figure 9.
